# AWD-stacking: An enhanced ensemble learning model for predicting glucose levels

**DOI:** 10.1371/journal.pone.0291594

**Published:** 2024-02-14

**Authors:** HuaZhong Yang, Zhongju Chen, Jinfan Huang, Suruo Li

**Affiliations:** 1 School of Computer Engineering, Jingchu University of Technology, Jingmen, Hubei, China; 2 School of Computer Science, Yangtze University, Jingzhou, Hubei, China; St Xavier’s Catholic College of Engineering, INDIA

## Abstract

Accurate prediction of blood glucose levels is essential for type 1 diabetes optimizing insulin therapy and minimizing complications in patients with type 1 diabetes. Using ensemble learning algorithms is a promising approach. In this regard, this study proposes an improved stacking ensemble learning algorithm for predicting blood glucose level, in which three improved long short-term memory network models are used as the base model, and an improved nearest neighbor propagation clustering algorithm is adaptively weighted to this ensemble model. The OhioT1DM dataset is used to train and evaluate the performance of the proposed model. This study evaluated the performance of the proposed model using the Root Mean Square Error (RMSE), Mean Absolute Error (MAE), and Matthews Correlation Coefficient (MCC) as the evaluation metrics. The experimental results demonstrate that the proposed model achieves an RMSE of 1.425 mg/dL, MAE of 0.721 mg/dL, and MCC of 0.982 mg/dL for a 30-minute prediction horizon(PH), RMSE of 3.212 mg/dL, MAE of 1.605 mg/dL, and MCC of 0.950 mg/dL for a 45-minute PH; and RMSE of 6.346 mg/dL, MAE of 3.232 mg/dL, and MCC of 0.930 mg/dL for a 60-minute PH. Compared with the best non-ensemble model StackLSTM, the RMSE and MAE were improved by up to 27.92% and 65.32%, respectively. Clarke Error Grid Analysis and critical difference diagram revealed that the model errors were within 10%. The model proposed in this study exhibits state-of-the-art predictive performance, making it suitable for clinical decision-making and of significant importance for the effective treatment of diabetes in patients.

## Introduction

Diabetes is a metabolic disorder involving inadequate insulin production or impaired function that causes changes in blood glucose levels (BGLs) [1]. The main types include Type 1 Diabetes (T1D), Type 2 Diabetes (T2D), and gestational diabetes [2, 3]. T1D stems from an autoimmune response that damages pancreatic β-cells [4], whereas T2D results from reduced insulin sensitivity or insufficient secretion [5]. Gestational diabetes can also develop during pregnancy [6]. Both hyperglycemia and hypoglycemia can cause complications, such as cardiovascular diseases, nephropathy, neuropathy, and retinopathy [7, 8]. Traditional diabetes management includes pharmacotherapy, diet, exercise, and self-monitoring. Pharmacotherapy involves oral medications and insulin injections, whereas dietary control regulates carbohydrate, fat, and protein intakes to maintain BGLs. Exercise improves insulin sensitivity and aids in glucose control. Self-monitoring, including blood and urine glucose tests, effectively helps patients manage BGLs [9]. The artificial pancreas (AP) is a closed-loop insulin delivery system that regulates blood glucose levels based on continuous glucose monitoring (CGM) data [10, 11], insulin infusion, and other available information [12].CGM technology monitors current blood glucose levels in real-time to assist T1D subjects in controlling blood glucose abnormalities [13–15].

In addition, predicting BGLs in real time is effective for T1D patients to avoid hypoglycemia, hyperglycemia, and related complications. Machine learning enables the real-time prediction of BGLs. Machine learning is crucial for predicting BGLs because it uses physiological data of patients and historical records to create predictive models [16]. These models can be trained to make predictions based on individual patient characteristics, enhancing accuracy and reliability. At the same time, machine learning can adjust the parameters of the prediction model in real time based on the prediction results and patient feedback, thus continuously optimizing the prediction results.

Furthermore, machine learning can dynamically adjust the parameters of the predictive model and continuously optimize the results. Deep learning excels at discovering data correlations, whereas ensemble learning fusion prediction results from multiple base estimators [17]. With advancements in computer hardware, ensemble deep learning models have become advanced solutions for BGL prediction. Combining predictions from multiple models improves the performance of the ensemble model, reducing the model variance and the risk of overfitting and increasing accuracy and robustness.

This study proposes an improved adaptive weighted deep ensemble learning (AWD-stacking) method for predicting the BGL of patients with type I diabetes mellitus (T1DM). First, continuous blood glucose data were pre-processed using Kalman filtering and double exponential smoothing. Second, improved LSTM models (bidirectional LSTM, StackLSTM, and vanillaLSTM) were used as base estimators in a stacking ensemble, with a linear regression model as the meta-model to predict BGL. The proposed method utilizes only BGL data from continuous glucose monitoring in the OhioT1DM clinical dataset, defining BGL prediction as a univariate time-series prediction. In the AWD-stacking method, multiple historical window techniques were proposed to predict the BGLs and a weighted similarity matrix was proposed with an affinity propagation clustering algorithm to weight the base estimators adaptively. The initial training and testing sets were integrated into the meta-estimator training, constructing an advanced BGL prediction method. The proposed method achieved a state-of-the-art BGL prediction accuracy for the OhioT1DM dataset. The key contributions of this study are as follows.

- This study proposes a new approach to BGL prediction using a deep ensemble neural network architecture based on a multi-history window technique.
- A more reliable BGL prediction model uses Kalman filtering and bi-exponential smoothing to mitigate sensor failures in the CGM readings.
- In this study, an improved propagation weighting algorithm for affinity clustering is proposed to enhance the connection strength between nodes by increasing the weight α, which makes it easier for similar nodes to cluster together.
- This study used stacking ensemble learning to predict blood glucose levels and improve prediction accuracy.

The rest of this paper is organized as follows. Section 2 discusses the relevant work on BGL prediction, highlighting the current status and limitations of the existing research. Section 3 provides an overview of the OhioT1DM dataset. Section 4 elaborates on our proposed method. Section 5 presents experimental results. Section 6 is a discussion. Section 7 provides a summary and future research directions.

## Related works

BGL prediction models are categorized into data-driven, physiological, hybrid, and fuzzy inference models [18]. Physiological models rely on mathematical representations of the human insulin-glucose feedback system for BGL prediction, offering strong interpretability and accuracy but requiring substantial input data. Hybrid models combine the strengths of both the physiological and data-driven models. Fuzzy inference models based on fuzzy logic theory were designed to address the uncertainty and fuzziness inherent in BGLs. In contrast, data-driven models don’t require extensive physiological parameters or specialized knowledge and can quickly establish accurate BGL prediction models. Consequently, most researchers have selected data-driven models for BGL prediction. In practical applications, the performance of data-driven models is comparable to that of the physiological models. The following sections will briefly discuss recent BGL prediction research from the past few years.

In 2020, Kezhi Li et al. [19] proposed a convolutional recurrent neural network for predicting BGLs, which was validated using the OhioT1DM dataset. The results demonstrated that the RMSE was 9.38 ± 0.71 mg/dL for a 30-minute PH and 18.87 ± 2.25 mg/dL for a 60-minute PH. The model exhibited strong competitiveness, ineffective prediction levels, and low time lag. In the same year, Zhu et al. [20] proposed a deep learning model based on a Dilated Recurrent Neural Network (DRNN) for predicting BGLs for the next 30 minutes. The RMSE of the proposed model is 18.9mg/dL. The experimental results indicated that the Dilated Recurrent Neural Network could effectively enhance the BGL prediction performance. In 2021, Rabby et al. [21] proposed a deep recurrent neural network model based on stacked long short-term memory (StackLSTM) for BGL prediction. They conducted experiments using the OhioT1DM (2018) dataset. To achieve a more accurate prediction, the authors considered that the BGL is affected by multiple factors and adopted an incremental learning strategy to learn other features, such as carbohydrate intake and high-dose insulin. The experimental results showed that the average RMSE of the StackLSTM model was 6.45 and 17.24 mg/dL for PHs of 30 and 60 minutes, respectively. The proposed method can predict BGL more accurately and help avoid abnormal BGLs in patients. In 2021, Dudukcu et al. [22] proposed a fusion model using LSTM, WaveNet, and Gated Recurrent Unit (GRU) for predicting BGLs. The experimental results showed that the proposed fusion model achieved RMSE values of 21.90 mg/dL, 29.12 mg/dL, and 35.10 mg/dL for PHs of 30, 45, and 60 minutes, respectively. The proposed algorithm was compared with state-of-the-art research results, and the best results were obtained. In 2021, Tena et al. [23] proposed two ensemble neural network-based models for predicting BGLs at three different PHs of 30, 60, and 120 minutes and compared their performance with ten recently proposed neural network models. The authors validated their models on the OhioT1DM dataset and found that the algorithm achieved an RMSE of 19.57±3.03 mg/dL for a PH of 30 minutes and an RMSE of 34.93±5.28 mg/dL for a PH of 60 minutes. In 2022, Yang et al. [24] proposed a personalized multivariable BGL prediction, an independent channel deep learning framework. The autonomous channel network in the framework learns representations from the input variables based on variable interconnected time-varying scales and domain knowledge, with a reasonable sampling period and sequence length, effectively avoiding input information redundancy and incompleteness. The authors validated the framework using the clinical dataset OhioT1DM, and the results showed that the RMSE was 18.930±2.155 mg/dL and the mean absolute relative difference (MARD) was 9.218±1.607% when the PH was 30 minutes. Compared with other BGL prediction methods, such as support vector regression (SVR), long short-term memory network (LSTM), dilated recurrent neural network (DRNN), temporal convolutional network (TCN), and deep residual time series network (DRTF), the proposed method achieved the best prediction performance for BGL. In 2023, Shuvo et al. [25] proposed a personalized blood glucose prediction model based on deep learning using a method that integrates multi-task learning (MTL). The authors validated the proposed model using the OhioT1DM dataset and conducted a detailed analysis and clinical evaluation using the RMSE, MAE, and Clarke Error Grid Analysis (EGA). The experimental results showed that the proposed algorithm achieved an RMSE of 16.06±2.74 mg/dL and an MAE of 10.64±1.35 mg/dL for a PH of 30 minutes, and an RMSE of 30.89±4.31 mg/dL and an MAE of 22.07±2.96 mg/dL for a PH of 60 minutes. Table 1 summarizes related research on blood glucose prediction using the OhioT1DM dataset.

**Table 1:** Related research on BGL prediction using the OhioT1DM dataset.

Year	Authors	Method	Dataset	RMSE (mg/dL)	
				PH = 30	PH = 60
2020	Li. K. H et al. [19]	CRNN	OhioT1DM (2018+2020)	9.38±0.71	18.87±2.25
2020	Zhu. T. Y et al. [20]	DRNN	OhioT1DM (2018+2020)	18.90	-
2021	Rabby. F. M et al. [21]	StackLSTM	OhioT1DM (2018)	6.45	17.24
2021	Dudukcu. H.V et al. [22]	LSTM, Wave-Net, GRU	OhioT1DM (2018+2020)	21.90	29.12
2021	Tena. F. U et al. [23]	ENN	OhioT1DM (2018+2020)	9.57±3.03	34.93±5.28
2022	Yang. T et al. [24]	AC-DLF	OhioT1DM (2018+2020)	18.93±2.15	-
2023	Shuvo. M. et al. [25]	D-MTL	OhioT1DM (2018+2020)	16.06±2.74	30.89±4.31

Note: CRNN: convolutional recurrent neural networks, DRNN: dilated recurrent neural network, StackLSTM: stacked long short-term memory, GRU: gated recurrent units, ENN: ensemble neural network, AC-DLF: autonomous channel deep learning framework, D-MTL: deep multi-task learning

The existing research on BGL prediction has not addressed sensor-reading errors, resulting in suboptimal predictions. This study proposed a new adaptive deep ensemble learning model, AWD-stacking, based on clinical data from T1D patients in the Ohi-oT1DM dataset to predict future BGLs for 30, 45, and 60 minutes. The AWD-stacking model employs an improved LSTM neural network as the base estimator in ensemble learning and an improved nearest-neighbor propagation clustering algorithm for adaptive weighting of the base estimators. The initial training and testing sets were integrated into the meta-model training, and linear regression was employed for the final prediction. The clinical accuracy of the proposed model was evaluated using the Clarke Error Grid Analysis (EGA). Compared to recent research and non-ensemble models, the AWD-stacking approach demonstrates superior accuracy, offering valuable guidance for clinical practice and helping prevent blood glucose abnormalities in patients with diabetic.

## Dataset

The proposed model was validated using the publicly available OhioT1DM datasets [26], which consisted of data from 12 T1D patients (seven males and five females) aged 20 to 80 years, using Medtronic 530G or 630G insulin pumps. The dataset included continuous glucose monitoring data recorded every five minutes over eight weeks for each patient, along with data on insulin, physiological sensors, and self-reported life events. In the OhioT1DM (2018) dataset, patients #559, #563, #570, #575, #588, and #591 wore Basis Peak fitness bracelets, while in the OhioT1DM (2020) dataset, patients #540, #544, #552, #567, #584, and #596 wore Empatica Embrace fitness bracelets for data collection. Each patient’s The last ten days of data were designated as the test set, and the remaining data were used as the training set. Table 2 summarizes the data, gender, age, training, and testing samples for the OhioT1DM dataset. Further information about the dataset can be found in the Data Availability section.

**Table 2:** Date, gender, age, training samples, and testing samples of the OhioT1DM dataset.

PID	Date	Gender	Age	Training samples	Testing samples
559	2018	female	40–60	10796	2514
563	2018	male	40–60	12124	2570
570	2018	male	40–60	10982	2745
575	2018	female	40–60	11866	2590
588	2018	female	40–60	12640	2791
591	2018	female	40–60	10847	2760
540	2020	male	20–40	11947	2884
544	2020	male	40–60	10623	2704
552	2020	male	20–40	9080	2352
567	2020	female	20–40	10858	2377
584	2020	male	40–60	12150	2653
596	2020	male	40–60	10877	2731

## Methods

This subsection presents the details of data pre-processing and the proposed model.

### Data preprocessing

**Fig 1:**
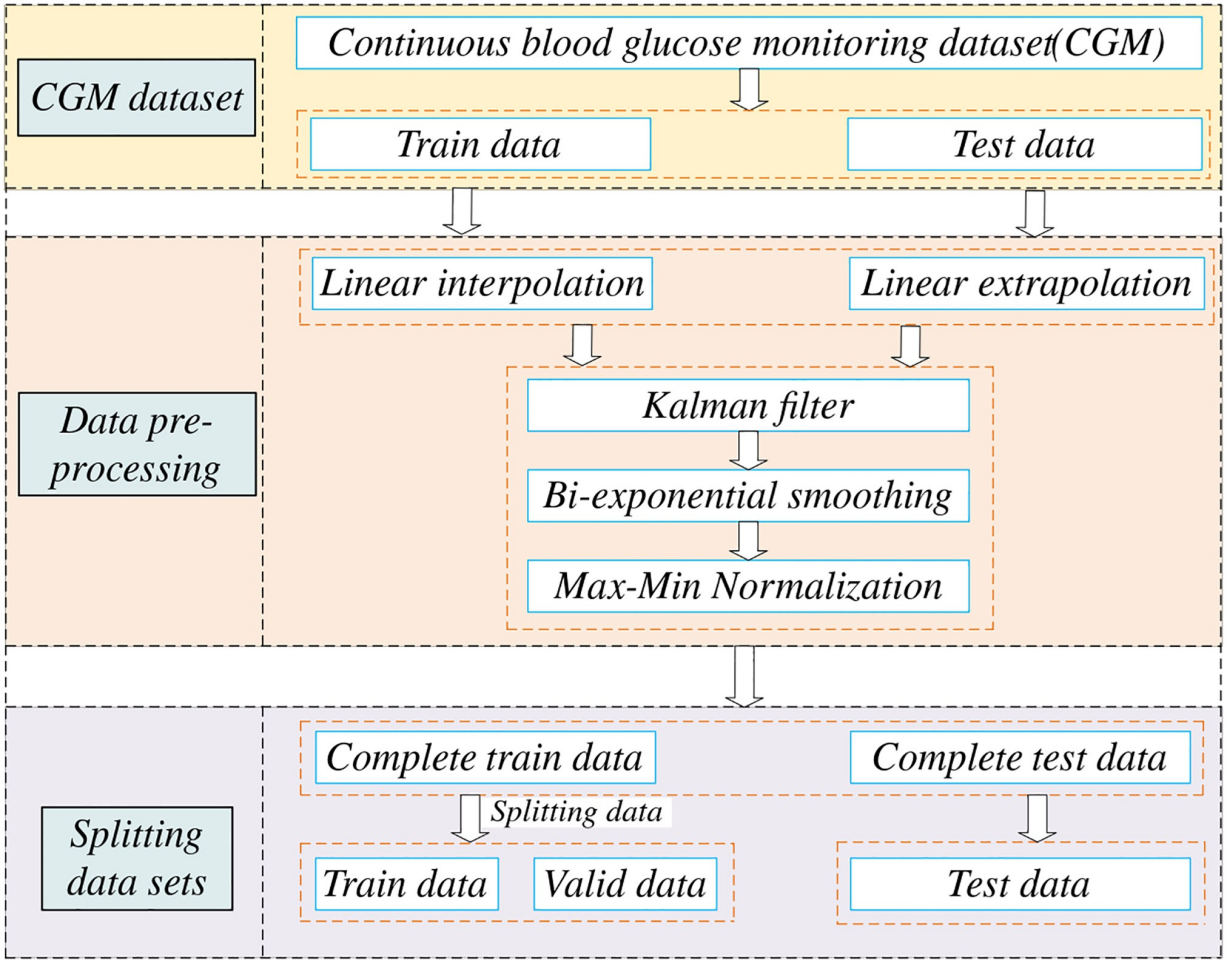
Data pre-processing sequence.

**Fig 2:**
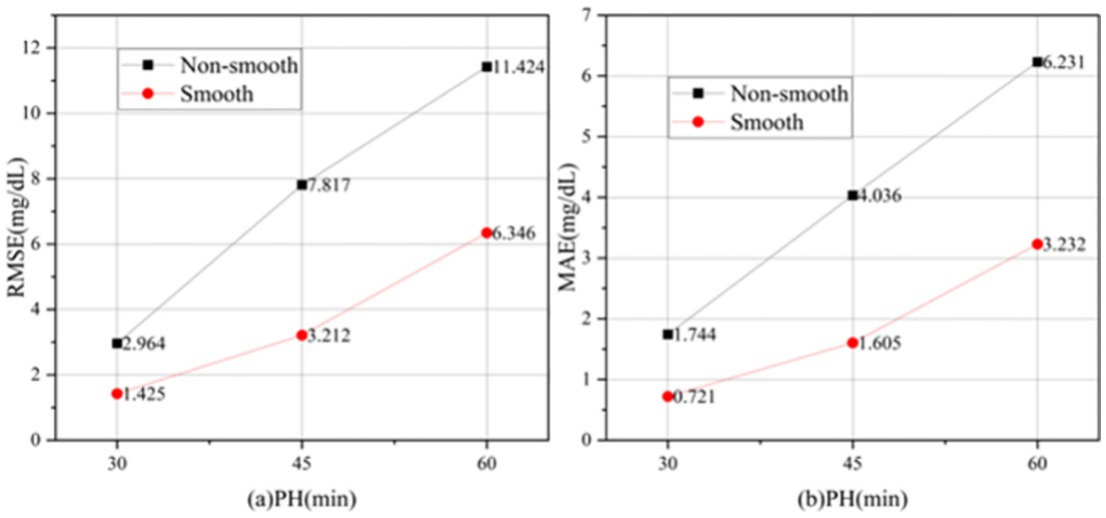
Experimental results of smooth and non-smooth(Black line represents unsmoothed experimental results, and the red line represents smoothed experimental results).

**Fig 3:**
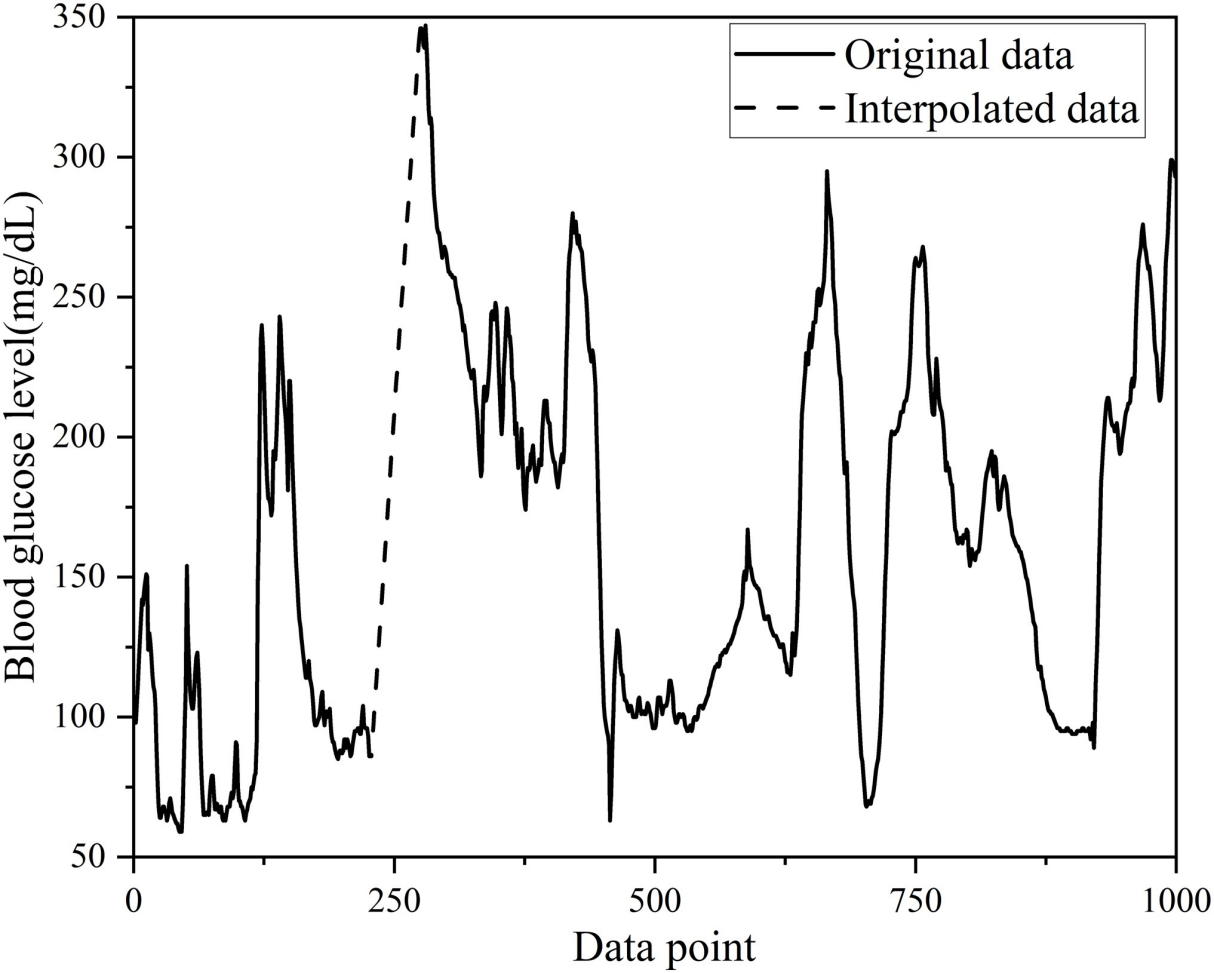
Original training set of patient #559 and the first 1000 data points after processing by linear interpolation.

This study utilized Kalman filtering [27–29] to address errors in the CGM readings, whereas double exponential smoothing was applied for data smoothing. The order of data preprocessing is shown in Fig 1. The steps were divided into six main steps. The first step is to collect the dataset. A historical dataset is used. The second step was to process the test and training sets separately, the training set using linear interpolation and the test set using linear extrapolation to ensure that the model did not observe future data. The third step was to use Kalman filtering for both the training and test sets to mitigate the errors caused by sensor readings. The fourth step was double exponential smoothing for the training and test sets to resolve data outliers. The experimental results of the smoothing process are shown in Fig 2. The fifth step was the normalization of the training and test sets. The sixth step was to divide the training set to 8:2. As a result, the BGL data were converted into a regular time series with 5-minute intervals, guaranteeing data completeness. After interpolation, Fig 3 illustrates the original training set and the first 1000 data points for patient #559.

The three pre-processing steps involved using a Kalman filter to process the blood glucose data. Because historical data are collected through sensors that measure interstitial fluid glucose levels, discrepancies exist between these readings and the actual BGLs. The Kalman filtering algorithm preprocesses the blood glucose data, yielding processed data that more closely correspond to the blood glucose values in the bloodstream. A brief overview of the Kalman filtering algorithm is provided below.

The Kalman filter is a state estimation filtering algorithm. Its core principle combines system state equations with observation equations and optimally estimates the system state values by minimizing the mean squared error. At each time step, the filtering process consisted of two stages: prediction (or forecasting) and updating (or correction). The prediction stage uses the system state equation to estimate the state value in the next time step. By contrast, the updating stage employs an observation equation to refine the predicted values and obtain a more accurate state estimation. The two-stage equations of the Kalman filtering algorithm are as follows.

Time update phase: System state equation, as shown in Eq 1.

$$x_{k}=Ax_{k-1}+Bu_{k-1}+w_{k-1}$$ (1)

where, ***x***_***k***_ denotes the state vector of the system at time k, A is the state transition matrix, ***u***_***k*−1**_ represents the input quantity, B corresponds to the input control matrix, and ***w***_***k*−1**_ indicates process noise.

The observation equation is as follows shown in Eq 2.


$$z_{k}=Hx_{k}+v_{k}$$ (2)

At time k, ***z***_***k***_ represents the observation vector, H corresponds to the observation matrix, and ***v***_***k***_ denotes observation noise.

The covariance update equation (time update) is given by Eq 3.


$${\bar{P}}_{k}=AP_{k-1}A^{T}+Q$$ (3)

In this case, ***Q*** represents the process noise covariance matrix.

Calculating the Kalman gain, used to balance the uncertainty between the predicted state estimates and observed data, is essential for determining informational advantage. The Kalman gain is given by Eq 4.

$$K_{k}={\bar{P}}_{k}H^{T}{\left(H{\bar{P}}_{k}H^{T}+R\right)}^{-1}$$ (4)

where, ***K***_***k***_ denotes the Kalman information gain, ***H***^***T***^ denotes the transpose of the observation matrix, and R denotes the observation noise covariance matrix.

The observed data were used in the observation update stage. *z*_*k*_ to update the state estimates, observed data, and Kalman gain were employed to refine the predicted state estimates. The equation for the update stage is shown in Eq 5.

$${\hat{x}}_{k}={\bar{x}}_{k}+K_{k}\left(z_{k}-H{\bar{x}}_{k}\right)$$ (5)

where, *z*_*k*_ is the observation vector at time k; $H{\bar{x}}_{k}$ denotes the predicted observation value derived from the predicted state estimate; and $z_{k}-H{\bar{x}}_{k}$ represents the observation residual.

The covariance update equation (observation update) uses Kalman gain to refine the predicted covariance matrix. The equation for updating the error-covariance matrix is shown in Eq 6.


$$P_{k}=\left(I-K_{k}H\right){\bar{P}}_{k}$$ (6)

Here, I represent the unit matrix.

**Fig 4:**
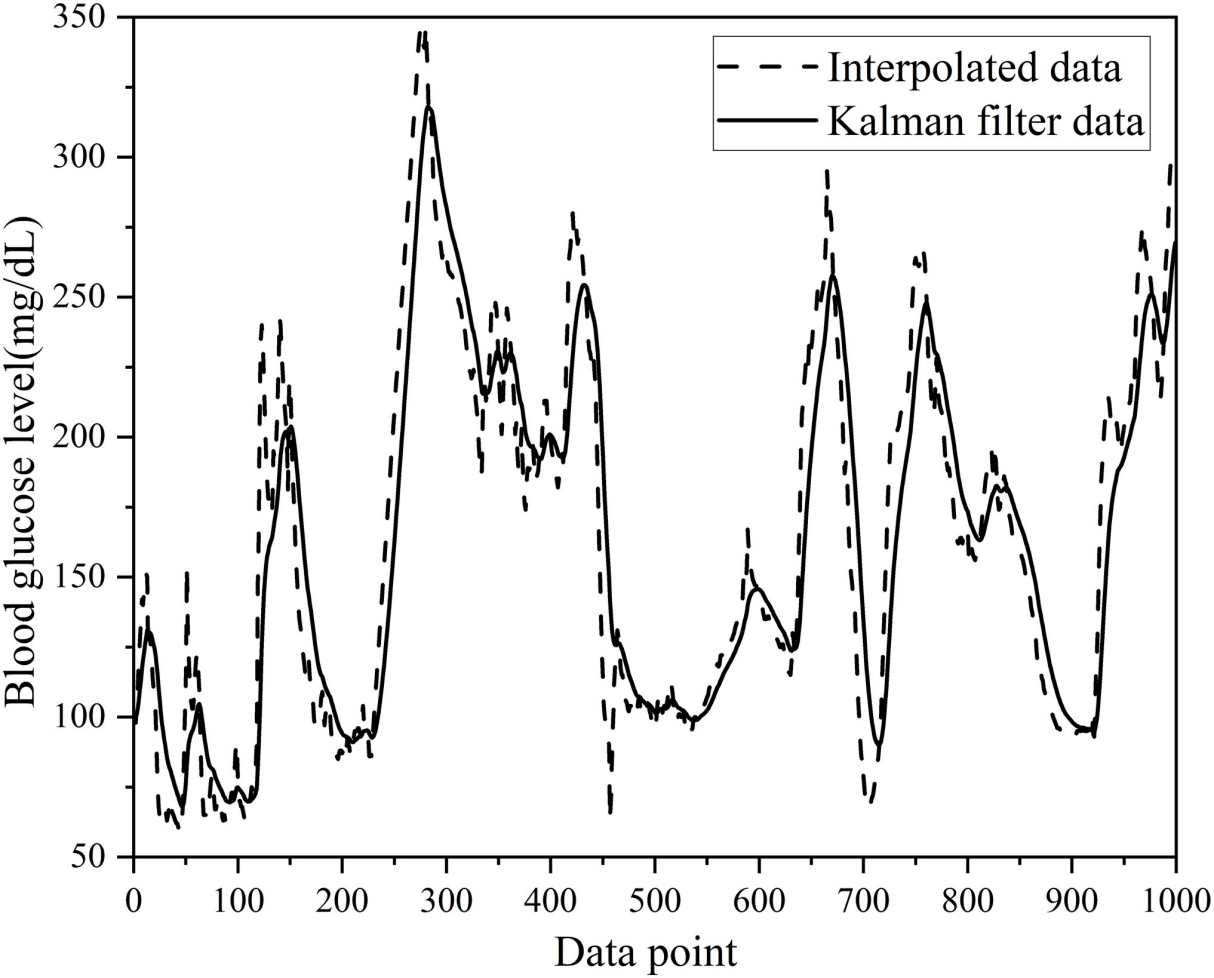
The first 1000 data points of the training set after the Kalman filtering process for patient #559.

After applying Kalman filtering, corrected BGL data were obtained. These data were utilized for the BGL prediction, ultimately enhancing the accuracy of the model. Fig 4 presents the first 1,000 data points in the training set of patient #559 after Kalman filtering.

**Fig 5:**
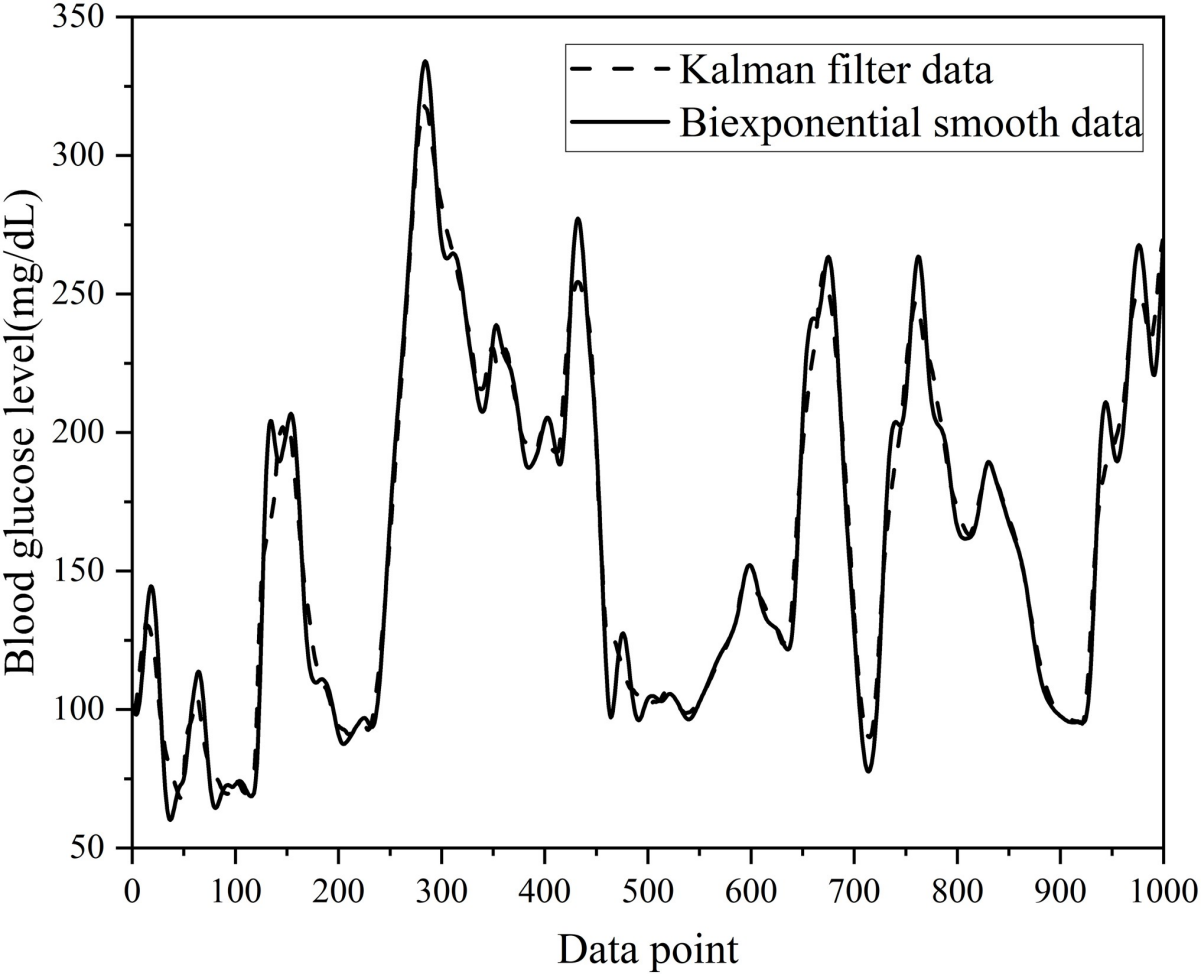
The first 1000 data points of the training set after double exponential smoothing of patient #559.

The four pre-processing steps involved applying a double exponential smoothing method [30] to the dataset. Because this forecasting task is a time-series prediction, this study employs double exponential smoothing techniques to process BGL data, resulting in increased continuity and stability and improved model prediction accuracy. Fig 5 displays the first 1,000 data points in the training set of patients with ID #559 after double exponential smoothing. A brief overview of the double exponential smoothing algorithm is presented below.

Double exponential smoothing primarily captures data level and trend components as they evolve. The following mathematical derivations illustrate the double exponential smoothing process.

The equation for smoothing the level component is shown in Eq 7.

$$L_{t}=\alpha Y_{t}+\left(1-\alpha \right)\left(L_{t-1}+T_{t-1}\right)$$ (7)

where, *L*_*t*_ represents the level component at time t, *Y*_*t*_ denotes the actual value at time t, *α* represents the level component smoothing coefficient (0< *α* <1), and *T*_*t*−1_ corresponds to the trend component at a given time.

The equation for smoothing the trend component is shown in Eq 8.


$$T_{t}=\beta \left(L_{t}-L_{t-1}\right)+\left(1-\beta \right)T_{t-1}$$ (8)

Here, *β* represents the trend component smoothing coefficient (0< *β* <1).

The formula for processing data through double exponential smoothing is given by Eq 9.

$$Y_{t}=L_{t}+T_{t}$$ (9)

where, *Y*_*t*_ denotes the smoothed data, and *L*_*t*_ and *T*_*t*_ represent the level and trend components at the current time, respectively. In this work, α and β are set to 0.1 and 0.5, respectively.

The fifth step involves converting the time series problem into a supervised learning task and transforming the time series into sequence samples. Lagged observations are inputs, whereas future observations are outputs [31]. This study used sliding window data with varying historical lengths of 6, 9, 12, and 18 as inputs, corresponding to 30, 45, 60, and 90 minutes of historical data. The outputs consist of 6, 9, and 12 data points, corresponding to PH of 30, 45, and 60 minutes, respectively. By integrating multiple historical window data, multi-scale features between the data points were adequately captured, enhancing the predictive performance of the proposed model.

The final step entails preprocessing the sequence sample dataset using the max-min normalization method and dividing the training set into a validation set and a training subset. Twenty percent of the data is divided as the validation set, with the remaining data allocated to the training subset. As for the test set, the dataset of the initial test set is used directly without further division.

### Proposed model

**Fig 6:**
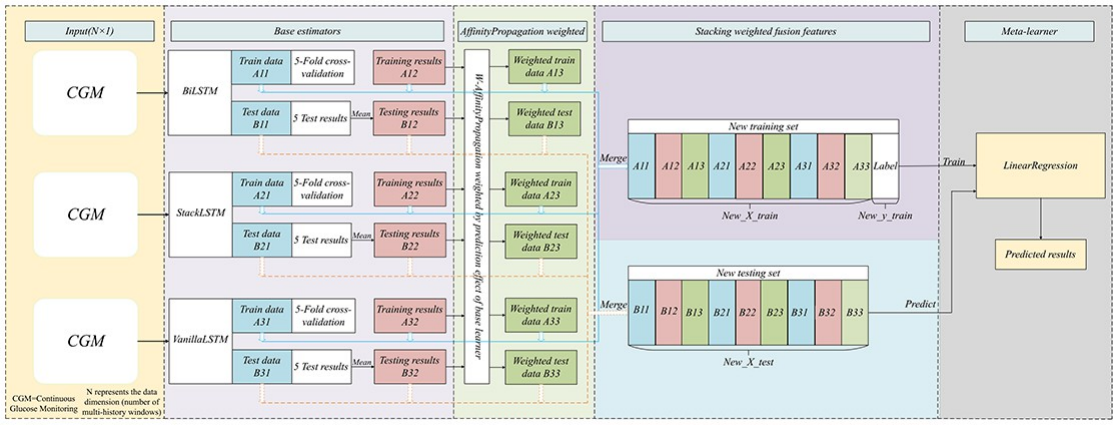
Diagram of the architecture of the AWD-stacking model.

This study used a linear model as the meta-model for BGL prediction owing to its simplicity and effectiveness. In addition, three improved time series forecasting models were introduced as the base estimators. An improved nearest neighbor propagation clustering algorithm was applied to weight the base estimators and enhance the prediction accuracy adaptively. Fig 6 displays the proposed deep ensemble learning architecture with adaptive weighting for BGL prediction. The subsequent sections discuss the three non-ensemble models and the AWD-stacking ensemble model.

#### Non-ensemble model

This study established a linear regression model and three improved LSTM non-ensemble models.

#### Linear regression

Owing to its simplicity, strong interpretability, wide applicability, high prediction accuracy, and ability to handle continuous variables [32], linear regression is applied as the meta-estimator for BGL prediction.

#### Bidirectional Long Short-Term Memory (BiLSTM)

**Fig 7:**
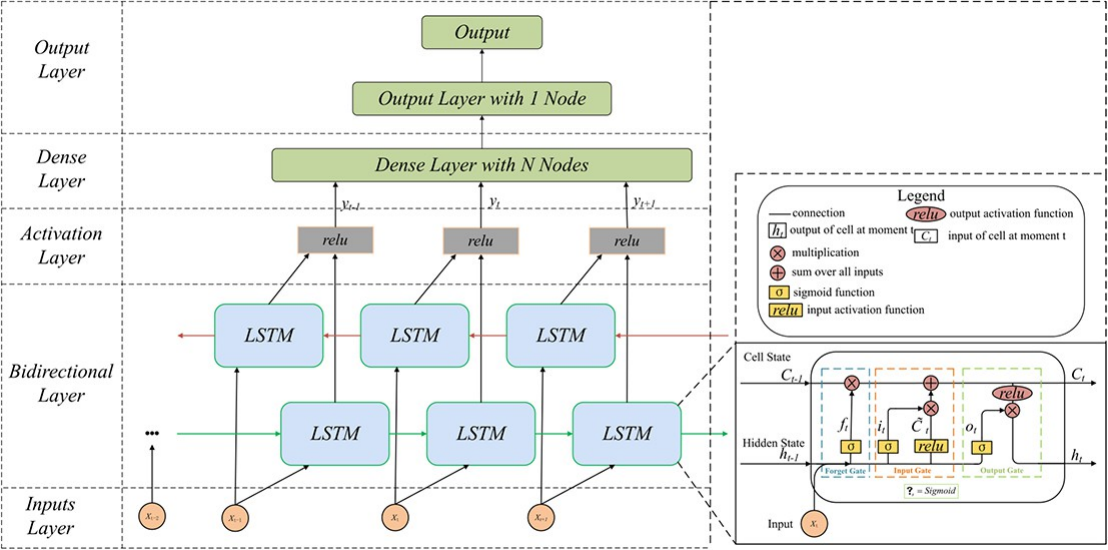
The architecture of the BiLSTM model for predicting BGLs.

A BiLSTM [33] network with vector output is used for multi-step prediction. To effectively predict time series data, BiLSTM processes inputs through forward and backward LSTM layers at each time step, concatenating the hidden states in both directions for the final output [34]. Fig 7 illustrates the architecture of the BiLSTM model for BGL prediction, consisting of 128 units, using the mean squared error as the loss function, a learning rate of 0.001, and the Adam optimizer. In the BiLSTM model, consisting of forward and backward LSTM, a single LSTM model cell possesses two states: the cell and hidden. The cell state *c*_*t*_ is used to transmit and update memory information, while *h*_*t*_ storing past information. A single LSTM unit encompasses three state gates: the forget gate *f*_*t*_, input gate *i*_*t*_, and output gate *o*_*t*_. The forward process equations for the BiLSTM model are as follows. Forward LSTM:

$$f_{t}=\sigma_{s}\left(W_{f}x_{t}+U_{f}h_{t-1}+b_{f}\right)$$ (10)

$$i_{t}=\sigma_{s}\left(W_{i}x_{t}+U_{i}h_{t-1}+b_{t}\right)$$ (11)

$$o_{t}=\sigma_{s}\left(W_{o}x_{t}+U_{o}h_{t-1}+b_{o}\right)$$ (12)

$$c_{t}=f_{t}⊙c_{t-1}+i_{t}⊙relu\left(W_{c}x_{t}+U_{c}h_{t-1}+b_{c}\right)$$ (13)

$$h_{t}=y_{t}=o_{t}⊙relu\left(c_{t}\right)$$ (14)

Here, *c*_*t*_ represents the cell state at the current time step, and *h*_*t*_ denotes the hidden state at that moment. The weight matrices and bias terms are denoted by W, U, and b, respectively. The input at the current time step is represented by *x*_*t*_, with *σ*_*s*_ representing the sigmoid activation function, relu representing the rectified linear unit activation function, and ⊙ indicating element-wise multiplication. Backward LSTM employs the same algorithm as forward LSTM, except that the input data and weight matrices are calculated in reverse order. Furthermore, the hidden states from each direction are concatenated by merging the results. Finally, the concatenated hidden states were processed using an activation function to yield the predicted outcomes.

#### Stack Long Short-Term Memory (StackLSTM)

**Fig 8:**
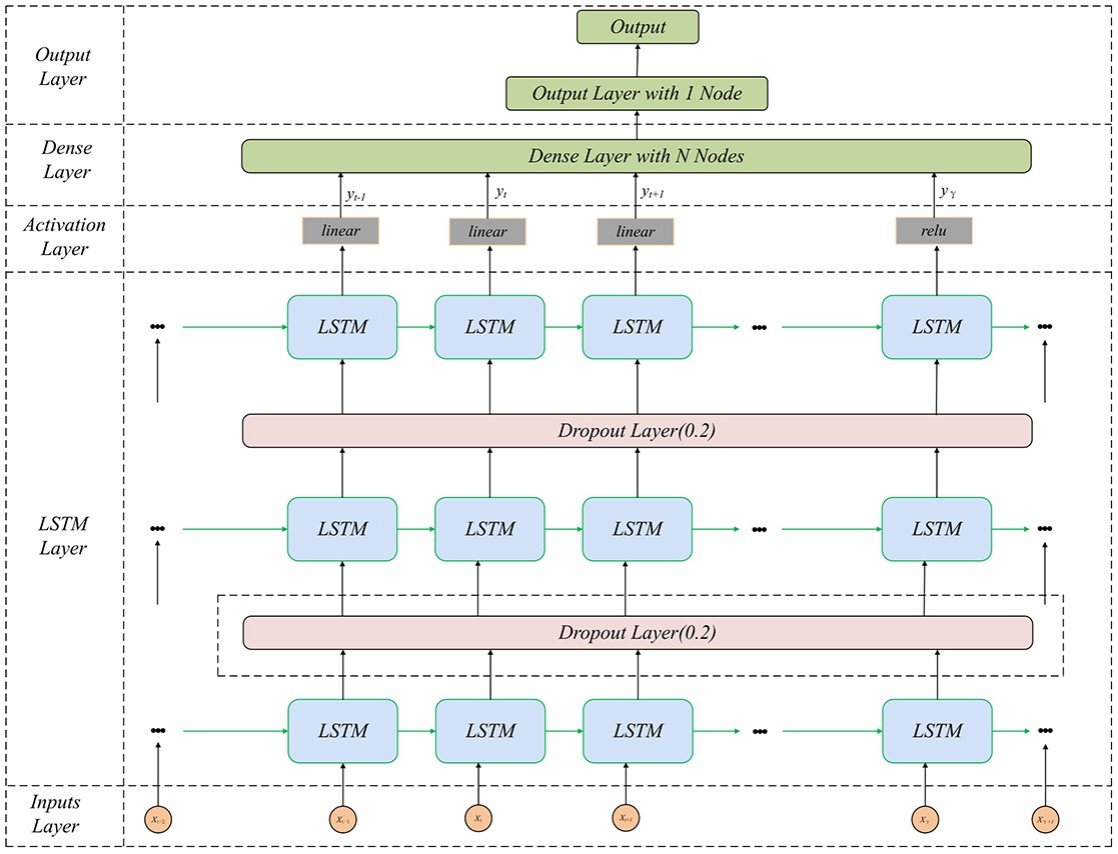
StackLSTM model architecture for predicting BGLs.

A deep neural network model based on LSTM [35], StackLSTM stacks multiple LSTM networks in a hierarchical structure to create a deeper model. The architecture for BGL prediction is depicted in Fig 8. The StackLSTM model has three LSTM layers with 128, 64, and 32 cell units in the first, second, and third layers. The output layer corresponds to future data points, using the mean squared error as the loss function, a learning rate of 0.001, and Adam as the optimizer.

**Fig 9:**
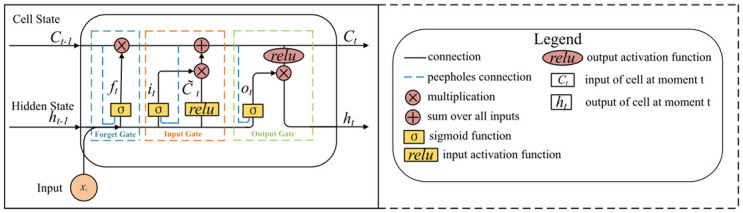
Architecture diagram of the VLSTM model.

Vanilla Long Short-Term Memory (VLSTM) is a recurrent neural network model [36] utilizing gating mechanisms to control the flow and retention of information. As depicted in Fig 9, the VLSTM model comprises an LSTM layer with 128 units, a fully connected (dense) layer with future data points as output nodes, the mean squared error as the loss function, a learning rate of 0.001, and the Adam optimizer. Peephole connections are added to the basic LSTM model to better handle long sequence data and capture long-distance dependencies.

The same training process ensured fairness in the non-ensemble model prediction results. The EarlyStopping callback function monitored the validation loss (*val_loss*) by setting the number of epochs to 10. The validation set evaluates the model performance and calculates the validation loss to prevent overfitting. During training, *epoch_size* was set to 500 and *batch_size* to 32, with *shuffle* and *verbose* set to True and 1, respectively. In non-ensemble models, the *return_sequences* parameter is set to *True*, indicating a multi-step time series prediction. The *ReLU* activation function was used, and the activation function for the dense layer was set to *linear*. In this study, because it is a multi-step, multi-history window time series prediction, the Dense is 6, 9, 12 and 18, respectively.

**Fig 10:**
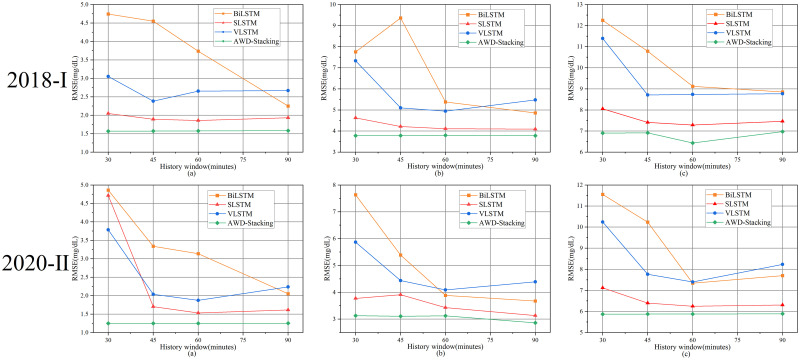
Results of the three base estimators and AWD-stacking model predictions using four historical windows of data for 30(a),45(b), and 60 (c) minute PH. Note: RMSE: Root mean square error; BiLSTM: Bi-directional long short-term memory; SLSTM: Stack long short-term memory; VLSTM: Vanilla long short-term memory;2018-I: In the OhioT1DM dataset, the data released in 2018 was used.2020-II: In the OhioT1DM dataset, the data released in 2020 was used Architecture diagram of the VLSTM model.

To investigate the effects of different history windows on the prediction results, the history windows were divided into 30, 45, 60 and 90 minutes. The average value is used as the final result. Fig 10(a) shows the results of the 30-minute prediction layer, Fig 10(b) shows the results of the 45-minute prediction layer, and Fig 10(c) shows the results of the 60-minute prediction layer. For the prediction ranges of 30, 45, and 60 minutes, the final hyperparameters selected for the best base learner BiLSTM, SLSTM, and VLSTM models are listed in Table 3.

**Table 3:** The base estimator hyperparameters.

Parameter	BiLSTM			SLSTM			VLSTM		
	PH:30	PH:45	PH:60	PH:30	PH:45	PH:60	PH:30	PH:45	PH:60
units	128	128	128	128	128	128	128	128	128
units	-	-	-	64	64	64	-	-	-
units	-	-	-	32	32	32	-	-	-
activation	relu	relu	relu	linear	linear	linear	linear	linear	linear
optimizer	Adam	Adam	Adam	Adam	Adam	Adam	Adam	Adam	Adam
lr	0.001	0.001	0.001	0.001	0.001	0.001	0.001	0.001	0.001
loss	mse	mse	mse	mse	mse	mse	mse	mse	mse
return_sequences	True	True	True	True	True	True	True	True	True

Note: lr: learning_rate; loss: loss function; mse: mean_squared_error

According to Fig 10, this study compares the six plots of the base learner predictions at 30, 45 and 60 minutes. As shown in the figure, three prediction range line graphs using four different history lengths produced different average RMSE values for the different datasets. Because different RMSEs exist between different history windows, the history length significantly affects the performance of the model. For the three prediction ranges, the RMSE decreased as the length of the history window data increased, indicating that using a more comprehensive history improved the prediction performance of the model. The AWD-stacking algorithm outperformed the other models with four data-history windows. This study considered that four different history windows affect the performance of the model. Therefore, the average of the four history windows was used as the final result in the following article to make the experimental results more accurate.

#### AWD-stacking ensemble model

Ensemble models are machine learning methods that enhance learning performance by integrating multiple base estimators into a powerful predictive model. By integrating the base estimators, overfitting is reduced, generalization ability is improved, and strong stability is exhibited when facing new data.

**Fig 11:**
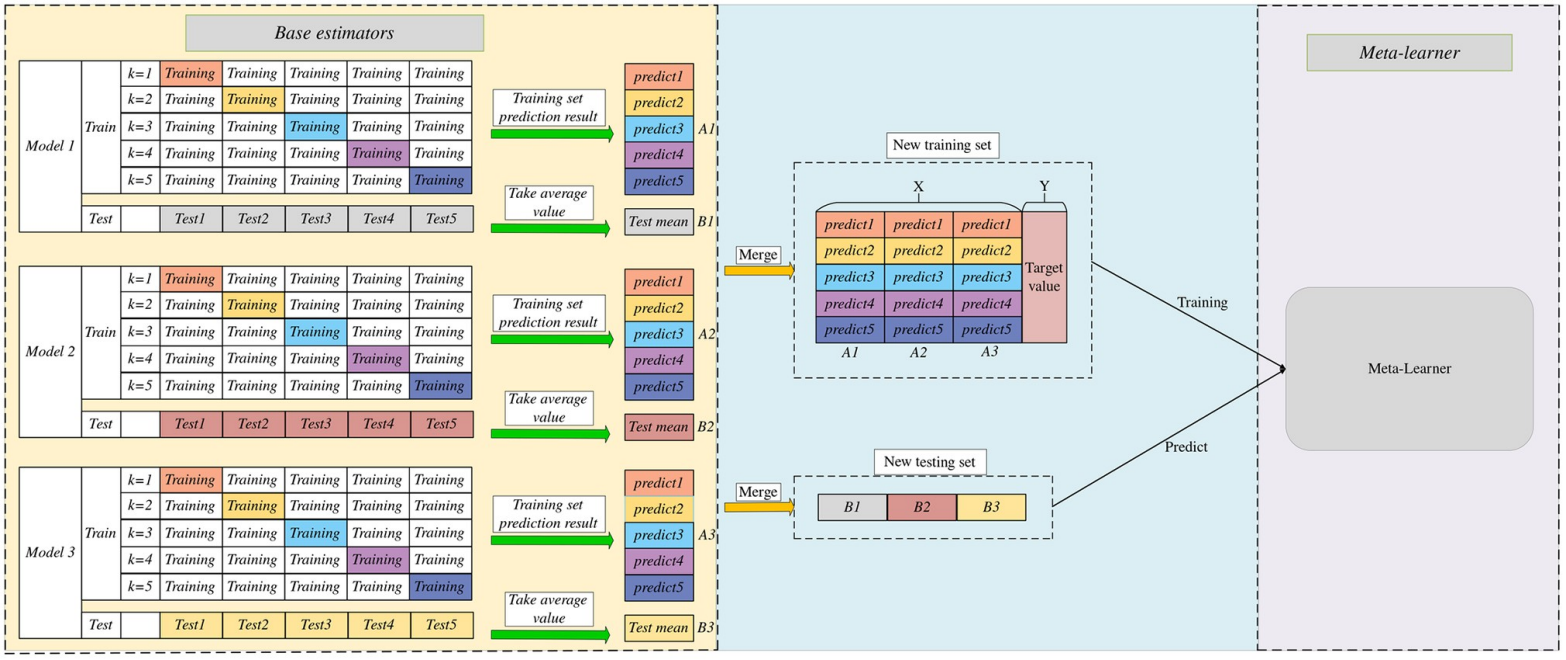
Stacking ensemble learning architecture diagram.

Stacking learning is an ensemble learning method that builds predictive models by training multiple base learners [37, 38]. In the stacking model, first-layer learners (base estimators) train on the original data, and their predictions serve as new feature inputs into the second-layer learner (meta-estimator). Combining this method allows stacking ensemble learning to capture multilevel relationships in the data and enhance the predictive performance. To prevent overfitting, this study employed 5-fold cross-validation. Fig 11 illustrates the architecture of stacking ensemble learning. In Fig 11, the base estimators represent part of the base learner. In this study, the base learner, 5-fold cross-validation was used for the training set to derive the final prediction result, training set prediction result,5-fold cross-validation was also performed for the test set, and finally, the average of the test set of each base model is used as the test set data of the meta-model, after certain combinations (as shown in Fig 11B1, 11B2 and 11B3). The results of the 5-fold cross-validation of the training set were combined into the training set of the meta-model. Specifically, the prediction results obtained by each base learner through the training set were combined, and the combined results are shown in the middle part (denoted by X) in Fig 11. The data were input into the meta-learner for the training set and prediction.

#### Meta-estimator

**Fig 12:**
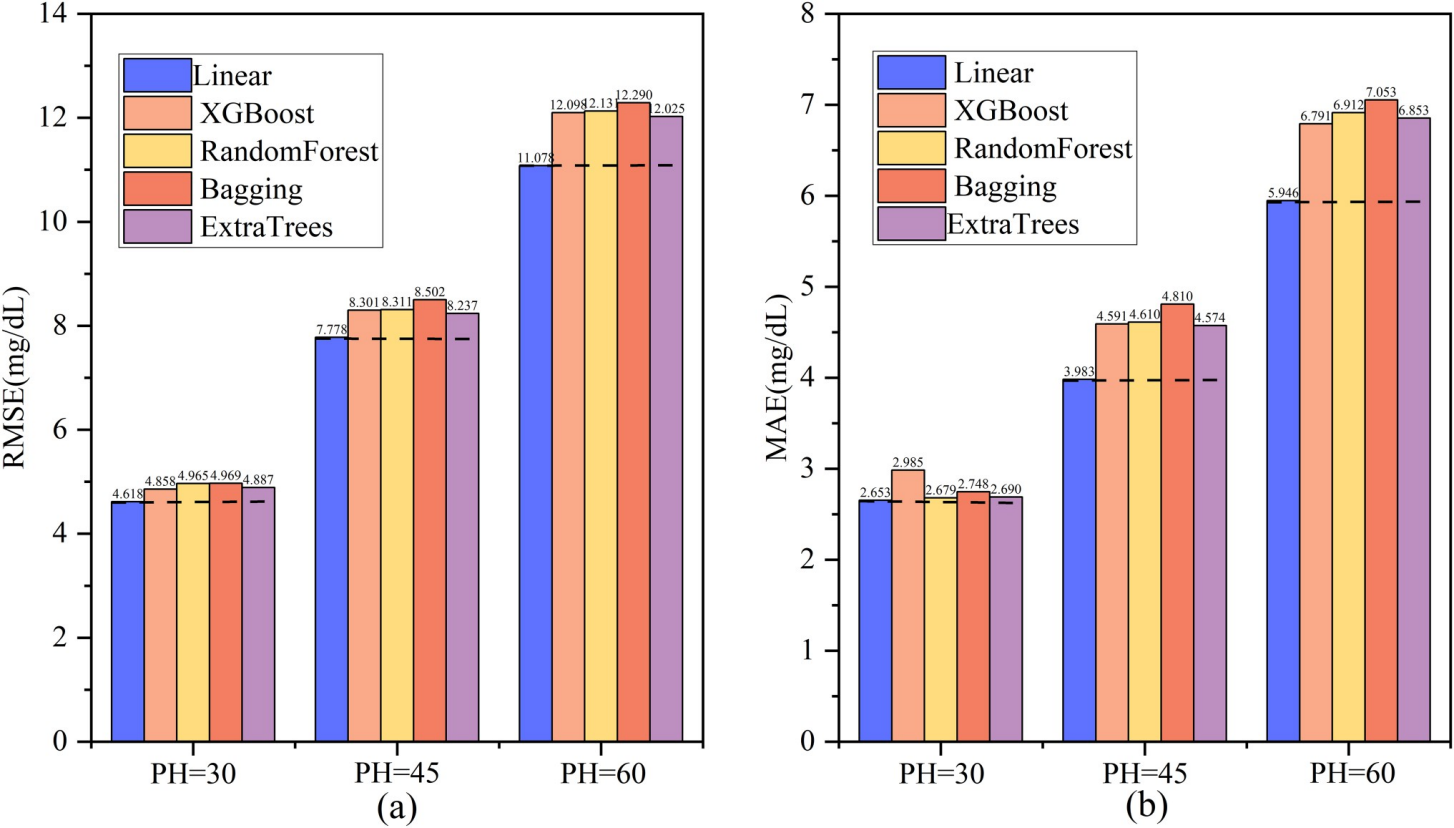
Experimental results of 5 meta-learners.

In ensemble learning, a meta-estimator is a learner that combines multiple base estimators. It uses base estimator predictions as inputs for further training, thereby enhancing generalization ability and performance. In this study, to compare the experimental results of different meta-estimators, five meta-estimators (Linear Regression, XGBoost, Random Forest, Bagging, and ExtraTrees) were used for experimental comparison. It should be noted that Bagging is a combination of regressors of bagging. This study used decision tree regression as this combiner for experiments. Since the best base learners in this study were BiLSTM, StackLSTM, and VanilaLSTM, the three models BiLSTM, StackLSTM, and VanilaLSTM are used as the base learners for integration learning in the experimental comparison of the meta-learners. The experimental results are shown in Fig 12. Fig 12(a) and 12(b) represent the experimental results of the evaluation indices RMSE and MAE for the five meta-models. The linear regression model as the meta-model has the best experimental effect, and the gap between the results of the linear meta-model and other meta-models increases as the prediction range increases, which verifies that the experimental linear meta-model is the best choice in this study.

#### Improved affinity propagation clustering algorithm

This study proposed a weighted similarity matrix affinity propagation (AP) clustering algorithm, integrating a similarity matrix and weight information to better represent relationships between data points and improve clustering performance. First, a weighted similarity matrix was constructed, where each element represents the similarity (distance) between the two base models. Next, the AP algorithm calculates each cluster center and members of each base model. Finally, the cluster center indices assign weights to each base model, which are then applied to weight the base models accordingly [39, 40]. The main steps and formulas for dynamically adjusting the weights of the base estimators using the improved AP-clustering algorithm are as follows.

The weighted similarity matrix is then calculated. The similarity matrix is calculated based on the distance between the data points, variance, and weight coefficients.


$$S_{i,j}=-{dis}_{i,j}-\alpha \left({var}_{i}+{var}_{j}\right)$$ (15)

$$dist_{i,j}=\sum_{k=1}^{n}(x_{i,k}-x_{j,k}{)}^{2}$$ (16)

$${var}_{i}=\frac{1}{n}\sum_{k=1}^{n}(x_{i,k}-{\overset{-}{x}}_{i}{)}^{2}$$ (17)

The similarity between data points i and j is represented by *S*_*i*,*j*_, where *dis*_*i*,*j*_ denotes the euclidean distance between them, the terms *var*_*i*_ and *var*_*j*_ represent the equations between data points i and j, and *α* is a weight coefficient. The feature dimension of the data points is denoted by ***n***, where ***x***_***i*,*k***_ and ***x***_***j*,*k***_ represent the values of data points i and j on the kth feature. Moreover, ${\overset{-}{x}}_{i}$ represents the average value of all the feature values for data point i.

An improved nearest neighbor propagation clustering algorithm is utilized to calculate the weighted variance sum of the clustering results, as shown in Eq 18.


$$w_{k}=\sum_{i=1}^{N}\frac{1}{C}\sum_{j\in c_{i}}(x_{j,k}-\mu_{i,k}{)}^{2}$$ (18)

In this context, *w*_*k*_ denotes the weighted variance sum of the kth feature, *N* denotes the weighted variance sum of the kth feature, and *C* denotes the number of clusters. The term *x*_*j*,*k*_ indicates the predicted result of the j-th sample on the k-th feature, *c*_*j*_ denotes the i-th cluster, and *μ*_*i*,*k*_ is the average value of the i-th cluster on the k-th feature.

The weight normalization is shown in Eq 19.


$$w_{k}=\frac{w_{k}}{\sum_{i=1}^{M}w_{j}}$$ (19)

Both the initial testing and training sets were separately incorporated into the meta-estimator for training to achieve optimal prediction results. At this stage, the training data for the meta-model include the initial training set, weighted training set, and predictions from the base estimators.

### Evaluation metrics

#### Root Mean Square Error (RMSE) and Mean Absolute Error (MAE)

This study evaluated the performance of regression models using two indicators: the Root Mean Square Error (RMSE) and Mean Absolute Error (MAE). Smaller RMSE and MAE values indicate better model performance. Eqs 20 and 21 depict the formulas for the RMSE and MAE, respectively.


$$RMSE=\sqrt{\frac{\sum_{i=1}^{n}(y_{i}-{\hat{y}}_{i}{)}^{2}}{n}}$$ (20)

$$MAE=\frac{1}{n}\sum_{i=1}^{n}\left|y_{i}-{\hat{y}}_{i}\right|$$ (21)

Here, *y*_*i*_ represents the actual value of the i-th sample, ${\hat{y}}_{i}$ denotes the predicted value, and n represents the sample size.

#### The Matthews correlation coefficient

The Matthews Correlation Coefficient (MCC) [41] is a model classification performance assessment metric. BGLs were categorized as low (BGL<70 mg/dL), normal (70 mg/dL ≤BGL<126 mg/dL), and high (BGL≥126 mg/dL) according to the International Federation of Diabetes (IDF). Hypoglycemia and hyperglycemia were defined as adverse events, whereas normal glucose levels were defined as normal. The results of the regression model predictions were converted to classification labels. The confusion matrix is a metric used to evaluate the performance of classification models. True Positive (TP) represents the number of samples with predicted and actual adverse events. True Negative (TN) refers to samples predicted and actual as normal events, False Positive (FP) denotes samples predicted as adverse events but normal, and False Negative (FN) indicates samples predicted as normal events but actually adverse events. Eq 22 presents the MCC calculation formula.


$$MCC=\frac{\left(TP*TN\right)-\left(FP*FN\right)}{\sqrt{\left(TP+FP\right)\left(TP+FN\right)\left(TN+FP\right)\left(TN+FN\right)}}$$ (22)

Error Grid Analysis (EGA) [37, 38] evaluates BGL predictions as a clinical indicator by comparing the actual measurements with the predicted values. The predicted results were divided into five regions (A, B, C, D and E), and the meanings of each region are listed in Table 4.

**Table 4:** Meanings of each region in Clarke EGA.

Regions	Implication
A	Predicted values are close to actual values, with errors within ±20%. The model exhibits good accuracy.
B	Predicted values have some errors compared to actual values, but these errors do not impact patient treatment, with errors generally within the range of ±20% to ±30%.
C	Errors between predicted and actual values are significant, potentially leading to erroneous clinical decisions and increased patient treatment risks. Unsuitable for clinical decision-making.
D	Errors between predicted and actual values are very large, potentially causing serious risks and clinical errors to patients during validation.
E	Predicted values are in the opposite direction of the actual values, possibly resulting in life-threatening treatment mistakes. Fundamental improvements to the model are required.

## Experimental results

In this section, we present experimental results and configurations. The RMSE, MAE, and MCC were used as evaluation metrics for the models in the experiments. Ensemble and non-ensemble models were used to predict BGLs at 30, 45, and 60 minutes. The training and test sets of the ohiot1dm dataset were used for the model training and evaluation. The experiment was implemented on a desktop platform configured with an Intel Core i7-8700HQ CPU, 16GB DDR4 RAM, 256GB SSD, Nvidia Geforce GTX 1050Ti graphics card and Windows 10 Professional (version: 21H2). The programming languages used in the experiments were Python (version: 3.8.10), TensorFlow (version: 2.11.0), Keras (version: 2.11.0), scikit-learn (version: 1.1.1), Numpy (version: 1.23.2), pandas (version: 1.5.0) and other machine learning libraries. Because of the versatility of TensorFlow, seven base models were built using TensorFlow and Keras (BiLSTM, StackLSTM, and VanilaLSTM), and the scikit-learn library was used to build meta-model algorithms (e.g., linear regression) and perform 5-fold cross-validation. Numpy and pandas were used for preprocessing (e.g., missing values and outlier handling). The best experimental results can be obtained using the above platforms and libraries, and the constructed algorithms can be ported to other platforms (including the corresponding libraries) to achieve the portability of the algorithms.

### Non-ensemble models

This study used four different historical window data (30, 45, 60, and 90min corresponding to 6, 9, 12, and 18 data points) to capture multi-scale features between the data. Tables 5 and 6 show the BGL predictions of the four historical window datasets. The evaluation results for the 12 patients using the non-ensemble models are listed in S1 Appendix.

**Table 5:** Experimental results for the four historical window data in the 2018 dataset, with PHs of 30, 45, and 60 minutes.

PH	Model	HW = 30min			HW = 45min			HW = 60min			HW = 90min		
		RMSE	MAE	MCC	RMSE	MAE	MCC	RMSE	MAE	MCC	RMSE	MAE	MCC
30	BiLSTM	4.745	3.092	0.936	4.552	3.051	0.926	3.737	2.574	0.955	2.250	1.267	0.97
	SLSTM	2.047	1.344	0.976	1.893	1.266	0.965	1.859	1.188	0.976	1.933	1.217	0.981
	VLSTM	3.051	1.842	0.963	2.384	1.470	0.962	2.655	1.455	0.972	2.669	1.597	0.967
45	BiLSTM	7.758	5.079	0.901	9.361	6.495	0.877	5.378	3.26	0.948	4.863	2.875	0.938
	SLSTM	4.626	2.849	0.954	4.214	2.549	0.960	4.115	2.308	0.960	4.092	2.343	0.957
	VLSTM	7.334	4.513	0.936	5.102	3.003	0.939	4.942	2.817	0.941	5.480	3.269	0.935
60	BiLSTM	12.252	8.013	0.838	10.790	6.522	0.884	9.12	5.502	0.906	8.857	5.182	0.905
	SLSTM	8.055	4.620	0.920	7.410	4.102	0.905	7.289	4.075	0.931	7.463	4.212	0.925
	VLSTM	11.395	6.850	0.870	8.713	5.065	0.898	8.741	5.131	0.902	8.769	5.038	0.901

Note: HW means history window

**Table 6:** Experimental results for the four historical window data in the 2020 dataset, with PHs of 30, 45, and 60 minutes.

PH	Model	HW = 30min			HW = 45min			HW = 60min			HW = 90min		
		RMSE	MAE	MCC	RMSE	MAE	MCC	RMSE	MAE	MCC	RMSE	MAE	MCC
30	BiLSTM	4.861	3.492	0.922	3.338	2.147	0.95	3.137	2.283	0.952	2.053	1.305	0.969
	SLSTM	4.716	1.142	0.974	1.701	1.224	0.975	1.53	1.007	0.971	1.614	1.044	0.978
	VLSTM	3.784	2.425	0.944	2.039	1.281	0.968	1.874	1.112	0.969	2.237	1.347	0.968
45	BiLSTM	7.637	4.897	0.894	5.389	3.256	0.923	3.885	2.256	0.948	3.674	2.150	0.952
	SLSTM	3.772	2.348	0.945	3.913	2.526	0.949	3.431	2.044	0.954	3.132	1.832	0.960
	VLSTM	5.866	3.559	0.919	4.439	2.753	0.939	4.090	2.342	0.952	4.389	2.708	0.938
60	BiLSTM	11.557	7.325	0.818	10.234	6.443	0.860	7.342	4.133	0.910	7.700	4.544	0.904
	SLSTM	7.124	4.278	0.909	6.398	3.740	0.913	6.249	3.626	0.919	6.306	3.584	0.925
	VLSTM	10.241	6.296	0.848	7.764	4.522	0.905	7.399	4.124	0.903	8.233	4.622	0.893

Tables 5 and 6 compare three non-ensemble models with varying PHs and historical window data: Bidirectional Long Short-Term Memory (BiLSTM), Stacked Long Short-Term Memory (SLSTM), and Vanilla Long Short-Term Memory (VLSTM). The SLSTM model exhibited superior performance, as evidenced by the lower RMSE and MAE values and higher MCC values. It maintained better stability across different PHs and historical windows, while the BiLSTM and VLSTM models showed more significant fluctuations in prediction accuracy. The prediction accuracy declined as the time range increased, with the SLSTM model exhibiting the highest performance among the non-ensemble models.

### Ensemble models

Tables 7–9 display the assessment results of the AWD-stacking model for 12 patients with 30, 45, and 60-minute PHs, respectively.

**Table 7:** Prediction results for 12 patients on the AWD-stacking model (multi-history window data, PH 30 minutes).

Dataset	PID	HW = 30min			HW = 45min			HW = 60min			HW = 90min		
		RMSE	MAE	MCC	RMSE	MAE	MCC	RMSE	MAE	MCC	RMSE	MAE	MCC
2018	559	1.282	0.620	0.987	1.284	0.623	0.988	1.288	0.628	0.989	1.285	0.623	0.989
	563	1.741	0.794	0.981	1.730	0.791	0.981	1.732	0.791	0.982	1.737	0.793	0.982
	570	1.100	0.558	0.993	1.096	0.557	0.967	1.096	0.554	0.993	1.101	0.554	0.993
	575	2.170	0.938	0.987	2.173	0.949	0.953	2.188	0.947	0.987	2.206	0.940	0.986
	588	1.301	0.699	0.979	1.326	0.707	0.987	1.314	0.700	0.980	1.323	0.708	0.979
	591	1.829	0.914	0.975	1.833	0.916	0.973	1.836	0.918	0.974	1.840	0.919	0.976
2020	540	1.047	0.552	0.984	1.044	0.552	0.983	1.046	0.552	0.984	1.046	0.552	0.984
	544	0.975	0.483	0.987	0.975	0.485	0.985	0.974	0.483	0.985	0.973	0.479	0.984
	552	0.935	0.403	0.994	0.935	0.414	0.994	0.934	0.401	0.995	0.937	0.416	0.994
	567	1.575	0.795	0.980	1.566	0.784	0.978	1.568	0.786	0.978	1.570	0.786	0.980
	584	1.824	0.937	0.973	1.830	0.936	0.971	1.824	0.933	0.965	1.827	0.938	0.971
	596	1.147	0.557	0.986	1.143	0.556	0.988	1.146	0.559	0.988	1.152	0.561	0.987

**Table 8:** Prediction results for 12 patients on the AWD-stacking model (multi-history window data, PH 45 minutes).

Dataset	PID	HW = 30min			HW = 45min			HW = 60min			HW = 90min		
		RMSE	MAE	MCC	RMSE	MAE	MCC	RMSE	MAE	MCC	RMSE	MAE	MCC
2018	559	3.166	1.560	0.969	3.164	1.555	0.972	3.196	1.579	0.969	3.146	1.554	0.961
	563	4.006	1.896	0.962	4.015	1.914	0.965	3.969	1.884	0.969	3.962	1.894	0.963
	570	2.705	1.376	0.987	2.689	1.370	0.985	2.709	1.372	0.987	2.705	1.358	0.981
	575	5.144	2.304	0.956	5.225	2.356	0.956	5.244	2.324	0.963	5.250	2.362	0.955
	588	3.295	1.725	0.958	3.268	1.716	0.958	3.299	1.734	0.957	3.226	1.700	0.957
	591	4.363	2.223	0.948	4.364	2.222	0.946	4.387	2.228	0.947	4.405	2.231	0.945
2020	540	2.692	1.428	0.955	2.668	1.415	0.954	2.674	1.416	0.955	2.696	1.417	0.954
	544	2.439	1.198	0.974	2.426	1.199	0.975	2.450	1.198	0.975	2.180	1.072	0.974
	552	2.396	1.027	0.980	2.383	1.037	0.981	2.386	1.031	0.980	2.373	1.037	0.979
	567	4.056	2.050	0.948	4.057	2.035	0.950	4.080	2.037	0.952	2.787	1.385	0.967
	584	4.405	2.259	0.939	4.399	2.267	0.939	4.388	2.253	0.940	4.362	2.231	0.940
	596	2.743	1.370	0.972	2.726	1.354	0.972	2.756	1.378	0.976	2.775	1.388	0.970

**Table 9:** Prediction results for 12 patients on the AWD-stacking model (multi-history window data, PH 60 minutes).

Dataset	PID	HW = 30min			HW = 45min			HW = 60min			HW = 90min		
		RMSE	MAE	MCC	RMSE	MAE	MCC	RMSE	MAE	MCC	RMSE	MAE	MCC
2018	559	5.953	3.024	0.945	5.956	3.016	0.954	6.071	3.050	0.956	5.884	2.954	0.950
	563	7.199	3.540	0.936	7.273	3.581	0.869	7.110	3.504	0.954	7.083	3.481	0.934
	570	5.038	2.611	0.969	5.014	2.584	0.928	5.034	2.580	0.963	5.092	2.606	0.969
	575	9.334	4.372	0.931	9.320	4.358	0.877	9.492	4.339	0.951	9.804	4.531	0.924
	588	6.017	3.174	0.923	6.047	3.174	0.926	3.003	3.176	0.932	5.961	3.147	0.934
	591	7.900	4.148	0.903	7.908	4.135	0.903	7.917	4.124	0.906	8.015	4.183	0.901
2020	540	5.144	2.790	0.915	5.135	2.779	0.917	5.132	2.750	0.915	5.147	2.771	0.916
	544	4.584	2.277	0.939	4.595	2.250	0.941	4.559	2.270	0.942	4.537	2.240	0.941
	552	4.564	1.991	0.963	4.615	2.008	0.960	4.708	2.017	0.961	4.616	1.981	0.960
	567	7.792	3.993	0.922	7.788	3.981	0.915	7.809	3.918	0.918	7.820	3.960	0.920
	584	8.125	4.229	0.884	8.123	4.236	0.879	7.998	4.151	0.891	8.087	4.202	0.887
	596	5.034	2.596	0.953	5.014	2.573	0.953	5.073	2.588	0.958	5.160	2.664	0.950

Table 7, which considers a 30-minute PH, shows RMSE values ranging from 0.934 to 2.206 mg/dL. Patient #575 from the 2018 data has the highest RMSE, while patient #552 from the 2020 data has the lowest RMSE. The MAE range is 0.403 to 0.938 mg/dL, with patient #575 from the 2018 data having the largest MAE and patient #552 from the 2020 data having the smallest. The MCC range is 0.965 to 0.994 mg/dL, with patient #552 from the 2018 data having the highest MCC and patient #584 from the 2020 data having the lowest. Through Table 7, using longer historical windows of data will have lower RMSE and MAE while the Matthews correlation coefficient of the model improves. Using the multi-history window technique, the model proposed in this study can capture the data trends and time dependence on different time scales, thus improving the prediction of the model.

Table 8, which considers a 45-minute PH, shows RMSE values ranging from 2.180 to 5.244 mg/dL. Patient #575 from the 2018 data has the highest RMSE, while patient #544 from the 2020 data has the lowest RMSE. The MAE range is 1.027 to 2.356 mg/dL, with patient #575 from the 2018 data having the largest MAE and patient #552 from the 2020 data having the smallest.

The MCC range is 0.939 to 0.987 mg/dL, with patient #570 from the 2018 data having the highest MCC and patient #584 from the 2020 data having the lowest. By utilizing multiple historical windows and the AWD-stacking algorithm, the information in the time series data can be better utilized and the accuracy of the forecasting model can be improved by combining data patterns at different time scales. Using multiple historical windows helps the model capture trends and changes at different time scales, while the AWD-stacking algorithm further improves the model’s predictive power by integrating data from multiple historical window sizes.

Table 9, considering a 60-minute PH, shows RMSE values ranging from 4.537 to 9.804 mg/dL. Patient #575 from the 2018 data has the largest RMSE, while patient #544 from the 2020 data has the smallest RMSE. The MAE range is 1.981 to 4.531 mg/dL, with patient #575 from the 2018 data having the highest MAE and patient #552 from the 2020 data having the lowest. The MCC range is 0.879 to 0.969 mg/dL, with patient #570 from the 2018 data having the highest MCC and patient #584 from the 2020 data having the lowest.

**Fig 13:**
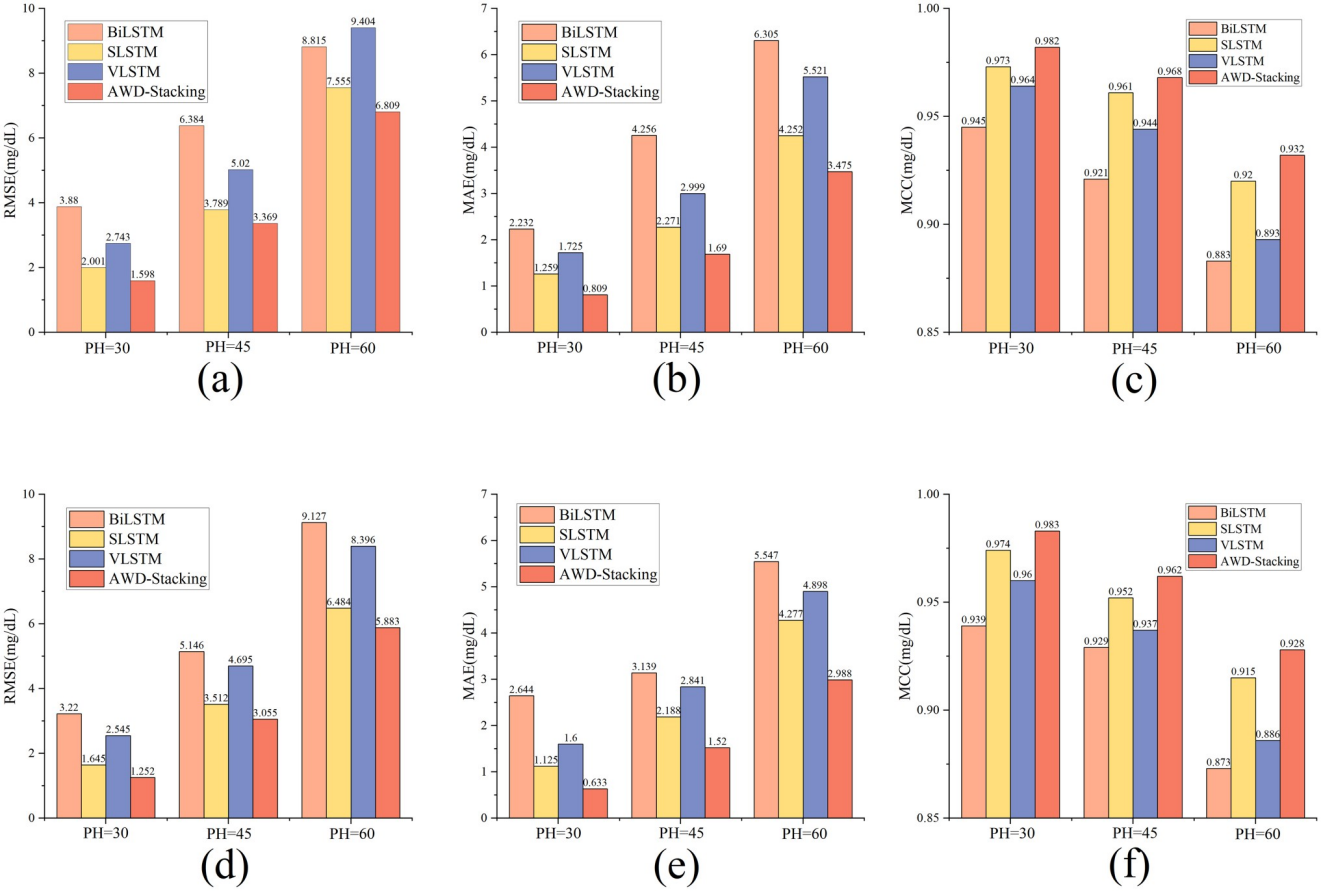
Presents the performance between the non-ensemble models and the AWD-stacking model. Note: (a), (b), and (c) represent the validation results of the models using 2018 data, while (d), (e), and (f) represent the validation results using 2020 data.

Tables 10 and 11 show the evaluation results of the non-ensemble models and AWD-stacking model for three PHs of 30, 45, and 60 minutes using three different evaluation metrics (RMSE, MAE, and MCC). Lower RMSE, MAE, and MCC values closer to 1 indicated better model performance. Observing the data in Table 10, the AWD-stacking model outperformed the SLSTM model for all PHs. Similar results can be observed in Table 11, where the AWD-stacking model surpasses the SLSTM model for all the PHs. Fig 13 illustrates this conclusion. The proposed AWD-stacking model in this study had significantly lower RMSE and MAE values than other non-integrated models at all three PHs for all patient data, indicating that the AWD-stacking model could predict blood glucose levels more accurately. In addition, the AWD-stacking model also showed advantages in (MCC values).

**Table 10:** Evaluation results of the non-ensemble model versus the AWD-stacking model for the three PHs of 30, 45, and 60 minutes in the 2018 data (in units of mg/dL).

PID	Model	PH = 30min			PH = 45min			PH = 60min		
		RMSE	MAE	MCC	RMSE	MAE	MCC	RMSE	MAE	MCC
559	BiLSTM	3.699	2.656	0.946	5.064	2.965	0.939	8.893	5.733	0.881
	SLSTM	1.887	1.403	0.969	3.601	2.166	0.961	6.457	3.638	0.925
	VLSTM	2.360	1.656	0.965	4.268	2.629	0.950	9.116	5.829	0.890
	AWD-stacking	1.285	0.896	0.988	3.168	1.562	0.968	5.966	3.011	0.951
563	BiLSTM	5.018	3.458	0.944	5.775	3.394	0.935	6.960	5.812	0.897
	SLSTM	1.974	0.979	0.977	4.299	2.324	0.964	7.735	4.154	0.928
	VLSTM	3.132	1.924	0.969	5.635	3.284	0.945	9.586	5.620	0.897
	AWD-stacking	1.735	0.792	0.981	3.988	1.897	0.964	7.166	3.526	0.933
570	BiLSTM	3.367	2.374	0.954	5.198	3.650	0.964	8.795	5.672	0.938
	SLSTM	1.813	1.383	0.977	3.148	1.968	0.977	5.650	3.272	0.940
	VLSTM	2.586	1.828	0.959	4.194	2.527	0.965	7.250	4.353	0.942
	AWD-stacking	1.239	0.608	0.990	2.702	1.369	0.985	5.044	2.595	0.957
575	BiLSTM	4.268	0.584	0.952	5.198	3.695	0.963	11.615	6.118	0.903
	SLSTM	2.377	1.209	0.977	3.148	1.968	0.977	9.940	5.271	0.921
	VLSTM	2.956	1.563	0.971	4.194	2.527	0.965	11.737	6.148	0.892
	AWD-stacking	2.184	0.942	0.977	2.702	1.369	0.985	9.487	4.400	0.921
588	BiLSTM	2.712	1.792	0.952	8.968	6.712	0.854	10.261	6.582	0.855
	SLSTM	1.864	1.278	0.972	3.847	2.449	0.950	6.782	3.971	0.916
	VLSTM	2.571	1.664	0.958	4.784	2.829	0.928	7.591	4.417	0.894
	AWD-stacking	1.316	0.703	0.979	3.272	1.719	0.958	5.257	3.168	0.929
591	BiLSTM	4.214	2.525	0.920	8.100	5.119	0.870	12.365	7.911	0.826
	SLSTM	2.093	1.299	0.967	4.695	2.753	0.939	8.763	5.206	0.891
	VLSTM	2.850	1.717	0.960	7.047	4.198	0.913	11.147	6.759	0.841
	AWD-stacking	1.834	0.917	0.974	4.380	2.226	0.947	7.935	4.148	0.903
AVG	BiLSTM	3.880	2.232	0.945	6.384	4.256	0.921	8.815	6.305	0.883
	SLSTM	2.001	1.259	0.973	3.789	2.271	0.961	7.555	4.252	0.920
	VLSTM	2.743	1.725	0.964	5.020	2.999	0.944	9.404	5.521	0.893
	AWD-stacking	1.598	0.809	0.982	3.369	1.690	0.968	6.809	3.475	0.932

**Table 11:** Evaluation results of the non-ensemble model versus the AWD-stacking model for the three PHs of 30, 45, and 60 minutes in the 2020 data (in units of mg/dL).

PID	Model	PH = 30min			PH = 45min			PH = 60min		
		RMSE	MAE	MCC	RMSE	MAE	MCC	RMSE	MAE	MCC
540	BiLSTM	4.198	3.152	0.917	5.282	3.420	0.902	8.191	5.161	0.851
	SLSTM	1.295	0.950	0.979	3.082	1.789	0.953	6.037	3.792	0.898
	VLSTM	2.179	1.438	0.963	4.176	2.651	0.927	7.806	4.916	0.857
	AWD-stacking	1.046	0.552	0.984	2.683	1.419	0.955	5.140	2.772	0.916
544	BiLSTM	1.198	3.152	0.917	3.830	2.276	0.947	6.885	3.917	0.901
	SLSTM	1.295	0.950	0.979	2.562	1.757	0.967	4.997	2.805	0.933
	VLSTM	2.179	1.438	0.963	3.337	1.888	0.959	6.542	3.821	0.908
	AWD-stacking	0.992	0.552	0.984	2.374	1.167	0.974	4.569	2.260	0.941
552	BiLSTM	2.941	2.242	0.967	4.401	2.601	0.956	6.665	3.404	0.921
	SLSTM	1.272	0.816	0.989	3.036	2.008	0.974	5.055	5.790	0.948
	VLSTM	2.182	1.324	0.979	4.058	2.457	0.970	6.583	3.485	0.928
	AWD-stacking	0.935	0.409	0.994	2.385	1.033	0.980	4.631	2.071	0.955
567	BiLSTM	4.087	2.676	0.939	6.662	4.019	0.903	12.167	7.322	0.846
	SLSTM	2.246	1.580	0.957	4.369	2.677	0.922	8.655	4.908	0.896
	VLSTM	3.228	1.886	0.947	6.620	3.846	0.906	10.821	5.999	0.877
	AWD-stacking	1.570	0.788	0.979	3.752	1.877	0.954	7.802	3.963	0.919
584	BiLSTM	4.143	2.736	0.932	5.906	3.562	0.920	12.612	8.188	0.804
	SLSTM	2.382	1.582	0.961	4.860	2.955	0.927	8.738	5.220	0.868
	VLSTM	3.249	2.025	0.939	5.848	3.539	0.908	12.030	7.453	0.809
	AWD-stacking	1.826	0.936	0.970	4.388	2.253	0.939	8.083	4.205	0.885
596	BiLSTM	2.775	1.910	0.963	4.795	2.960	0.948	8.244	5.290	0.912
	SLSTM	1.384	0.870	0.977	3.162	1.940	0.968	5.419	3.146	0.948
	VLSTM	2.254	1.490	0.971	4.135	2.662	0.952	6.597	3.715	0.936
	AWD-stacking	1.147	0.558	0.987	2.750	1.372	0.972	5.070	2.661	0.954
AVG	BiLSTM	3.220	2.644	0.939	5.146	3.139	0.929	9.127	5.547	0.873
	SLSTM	1.645	1.125	0.974	3.512	2.188	0.952	6.484	4.277	0.915
	VLSTM	2.545	1.600	0.960	4.695	2.841	0.937	8.396	4.898	0.886
	AWD-stacking	**1.252**	**0.633**	**0.983**	**3.055**	**1.520**	**0.962**	**5.883**	**2.988**	**0.928**

To demonstrate the superiority of the algorithm proposed in this study, four benchmarking models were added to the experiment, and the model proposed in this study had the best prediction effect by comparison, as shown in Part 2 of the S1 Appendix. The four added benchmarking models were convolutional neural networks-bidirectional long short-term memory (CBiLSTM), directional long short-term memory-attention (CBiLSTMA), multi-head-attention-bidirectional long short-term memory (MABiLSTM), and bidirectional long short-term memory-attention (BiLSTMA). A comparison of the average experimental results of the four benchmarking models with those of the proposed algorithm shows that the proposed model has the best results. The proposed model uses integrated learning, which can learn the advantages of each base learner and improve overall prediction results. Ensemble models are also an important direction for future research compared to individual learning models. Consequently, based on Tables 10 and 11, the proposed AWD-stacking model exhibits the highest accuracy and stability.

**Fig 14:**
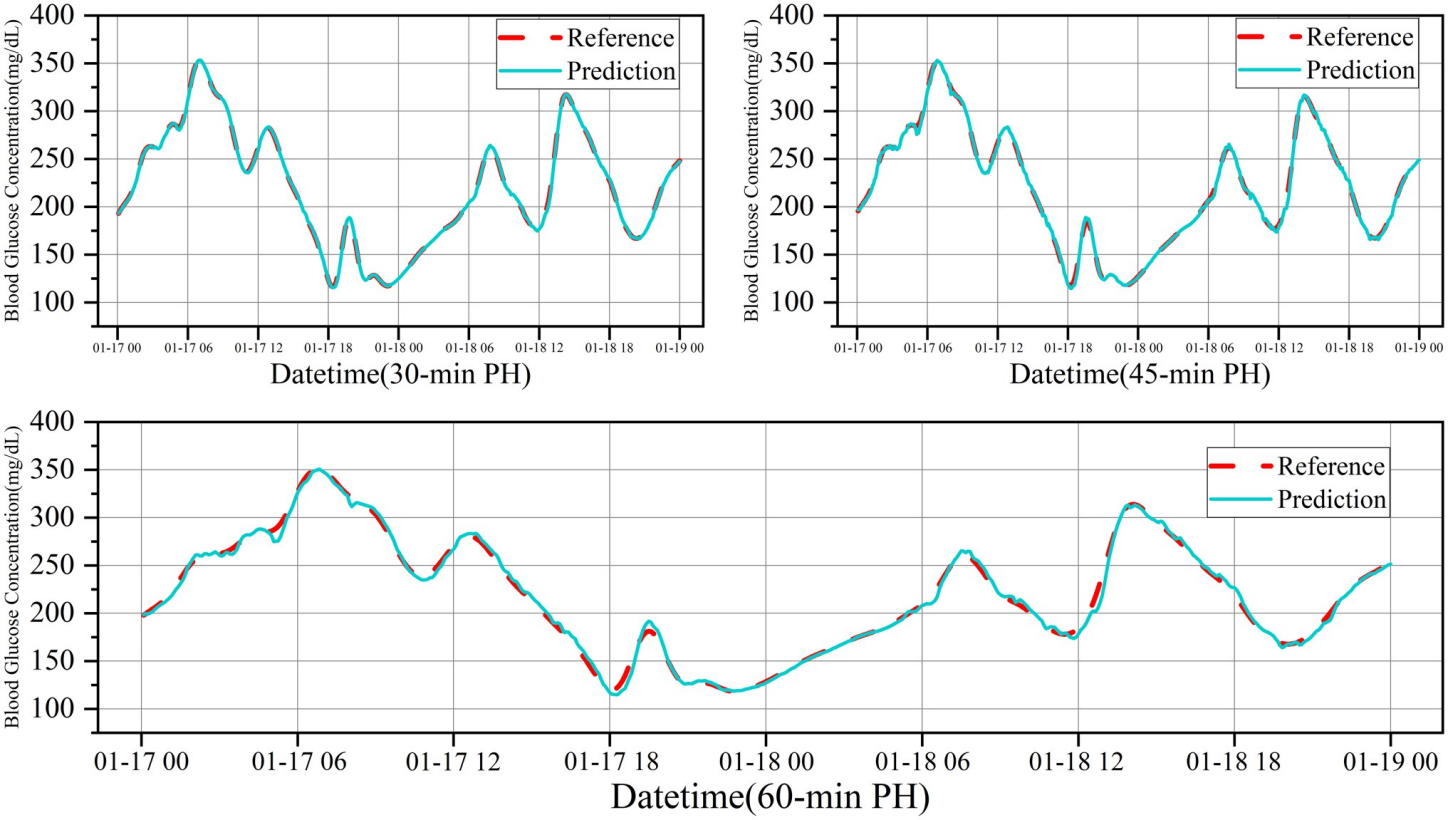
Glucose trajectory of patient #570 during 48 hours.

**Fig 15:**
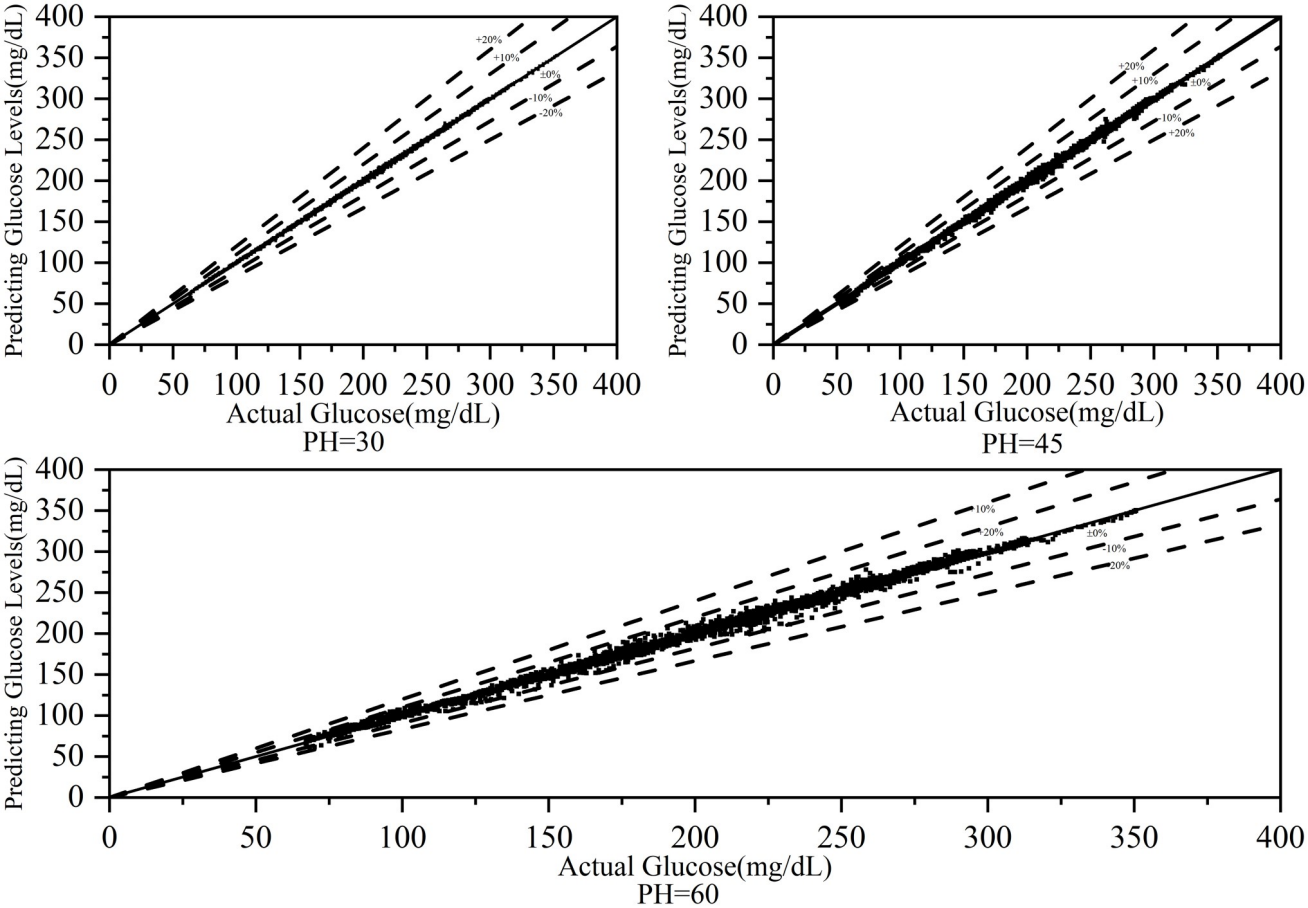
Error plot for patient #570.

Fig 14 depicts the glucose trajectory of patient #570 over 48 hours, showing a small discrepancy between the predicted and actual values, indicating the high accuracy and stability of the model. Fig 15 shows the error fitting plot for patient #570.

An error range between +10% and -10% signifies excellent prediction results, whereas a range between +20% and -20% indicates good results. For PHs of 30, 45, and 60 minutes, all predictions fell within the +10% to -10% range, demonstrating the excellent performance of the model.

**Fig 16:**
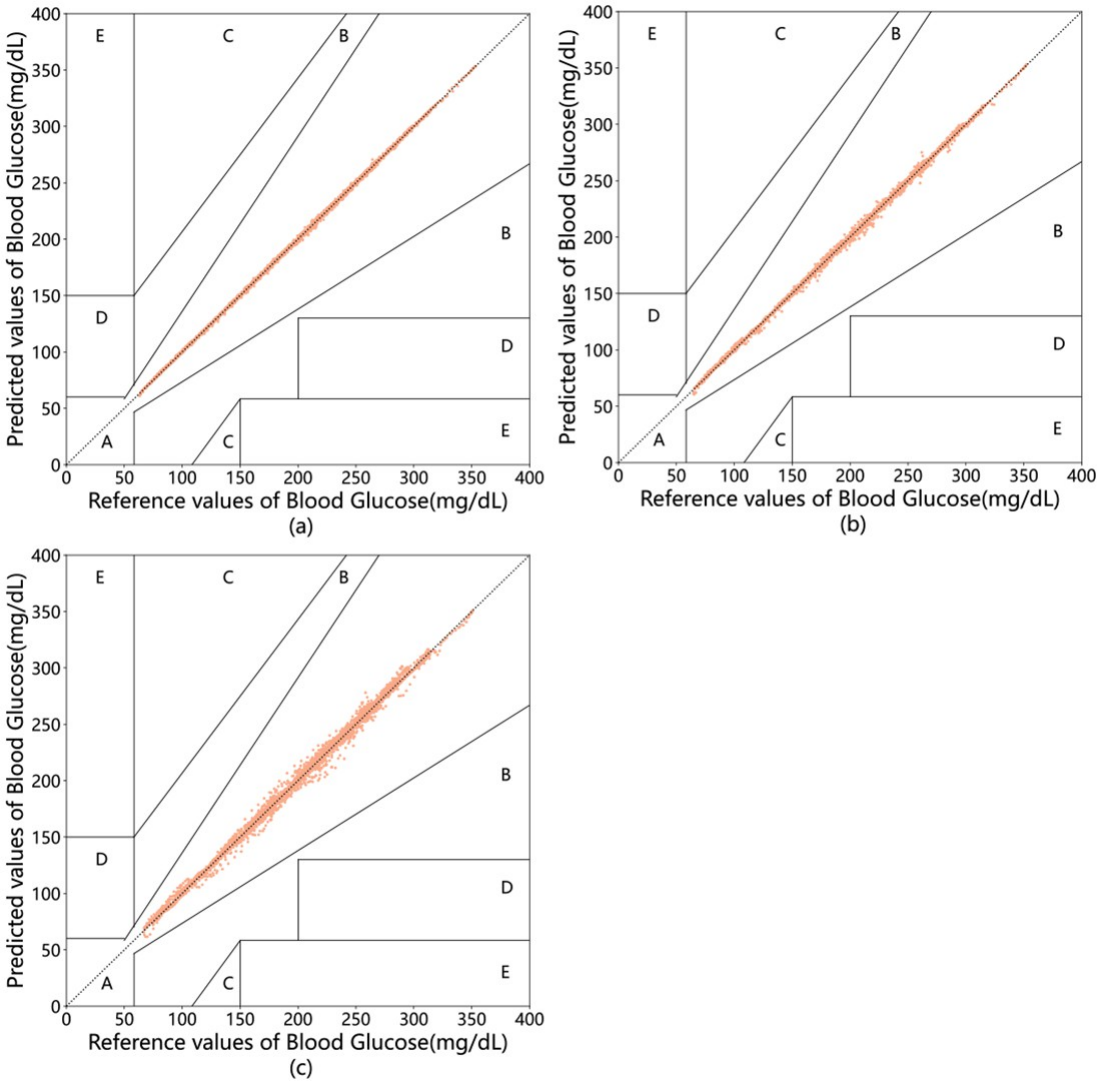
Clarke EGA plot for patient #570 at 30(a),45(b),60(c) minutes PH.

Clarke EGA plots use a higher point density to signify a better or worse model performance in specific areas [42]. Fig 16 shows the Clarke EGA plot for patient #570, demonstrating the performance of the proposed model in predicting BGLs. The data points are predominantly located in Zone A for PH of 30, 45, and 60 minutes, demonstrating the high accuracy and practical significance of the model in clinical settings.

### Statistical analysis

**Fig 17:**
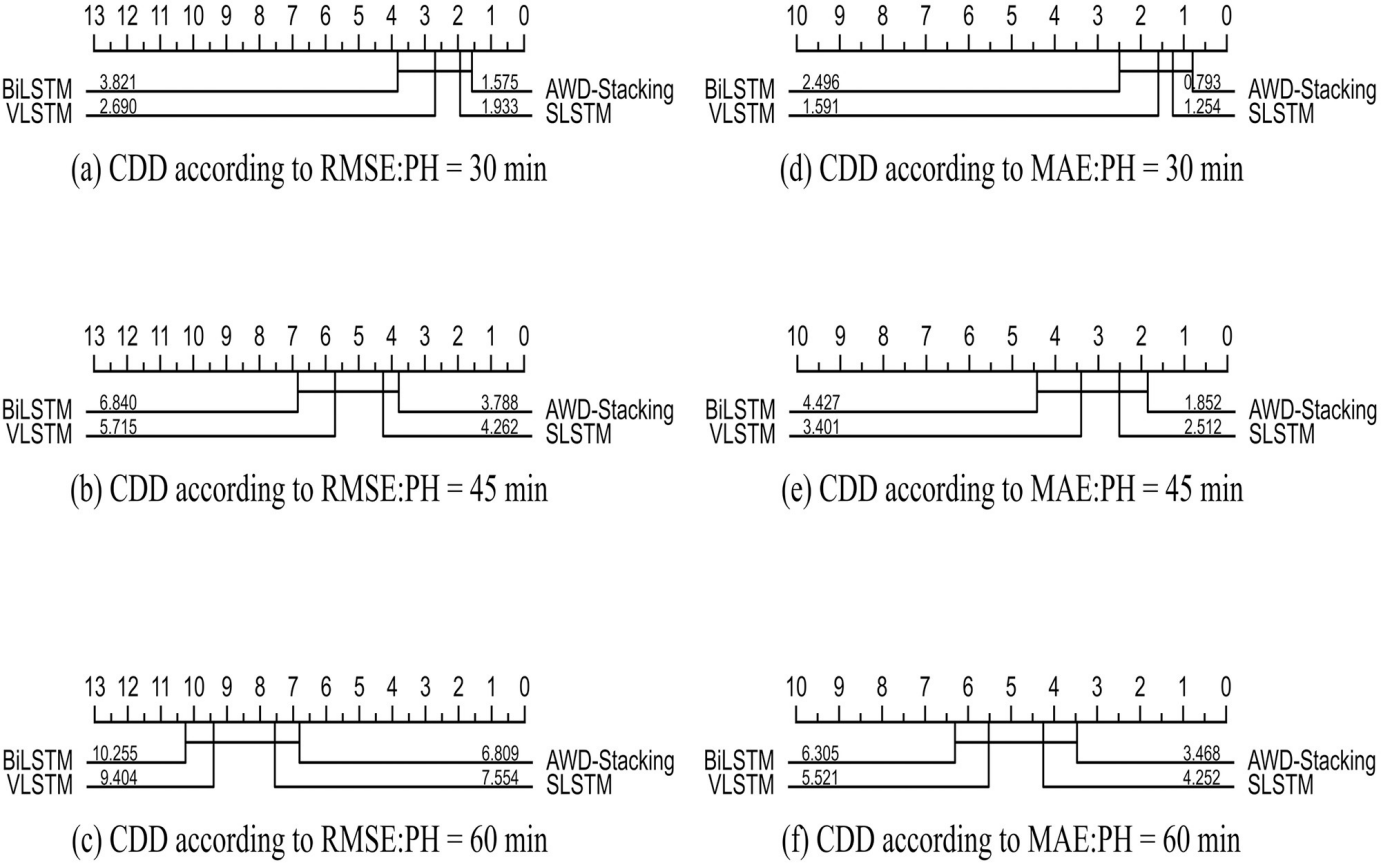
Critical difference diagrams for pairwise comparisons of all prediction models within the PH of 30 (a, d), 45 (b, e), and 60 (c, f) minutes.

**Fig 18:**
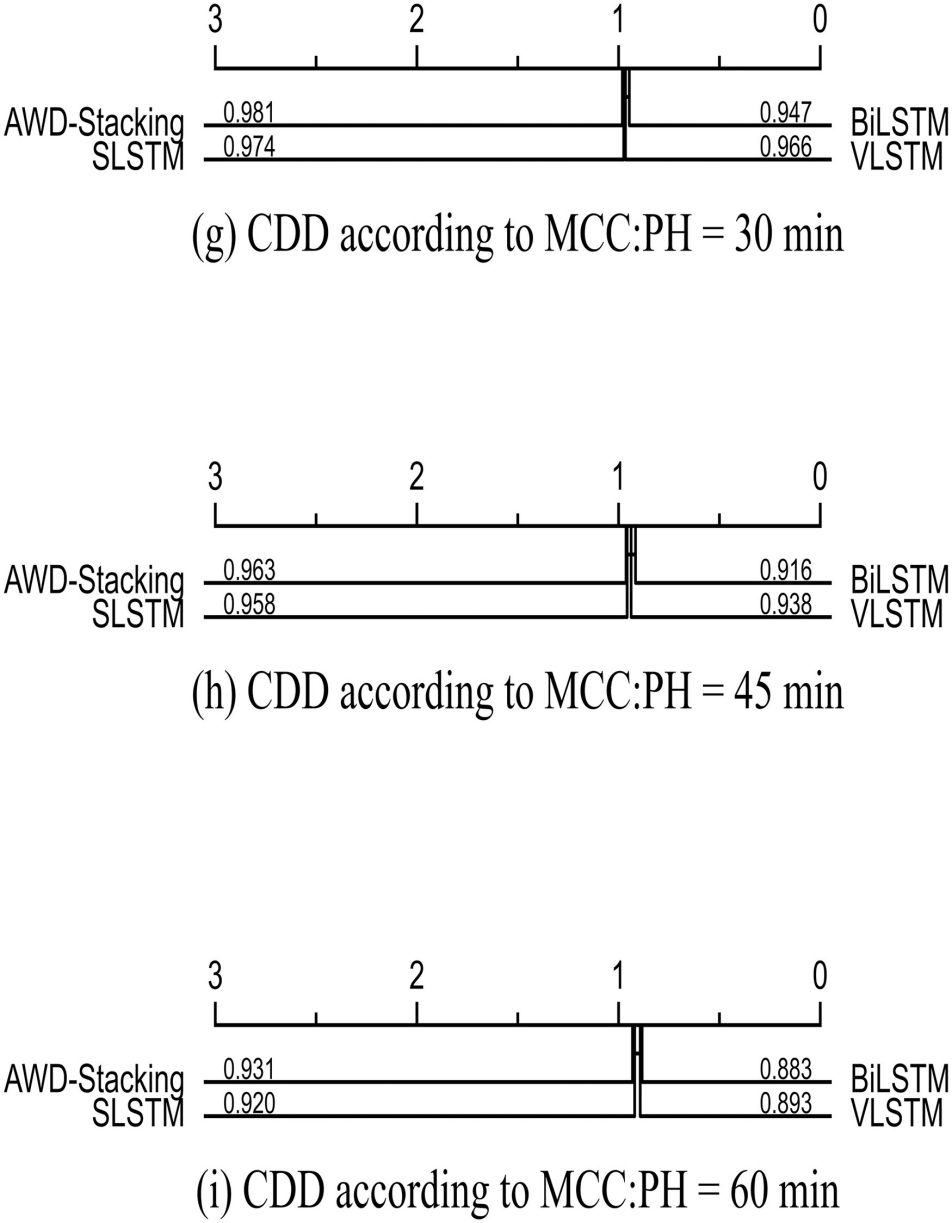
Critical difference diagrams for pairwise comparisons of all prediction models within PH of 30 (g), 45 (h), and 60 (i) minutes.

The statistical analysis results included the p-values of the Wilcoxon post-hoc test for two-by-two comparisons of all models and CDD values of the underlying learners. A visual overview of the future is available and the CDD plots are shown in Figs 17 and 18. A critical difference plot (CDD) was used to compare the performances of the different machine learning models. In the CDD, each point represents a model, the horizontal axis represents the ranking of the model, and the vertical axis represents the performance metric. Models positioned more to the left or right indicated superior performance. Figs 17 and 18 show the CDD validated using the 2018 data, with RMSE and MAE as evaluation metrics. As shown in Fig 17(a)–17(c), the RMSE of the AWD-stacking model are 1.575 mg/dL, 3.788 mg/dL and 6.809 mg/dL for pH values of 30, 45 and 60 min, respectively. In Fig 17(d)–17(f), for pH values of 30, 45 and 60 min, the RMSE of the AWD MAE for the Stacking model was 0.793 mg/dL, 1.852 mg/dL and 3.468 mg/dL, respectively. Fig 18 shows the main differences between all prediction models using the MCC evaluation metric on the 2018 dataset.

After statistical analysis, the experimental results of all models compared two by two according to each evaluation metric are presented in detail in Tables 12–14. The ensemble models were significantly better than non-ensembled models, with no significant differences in internalities. This study uses p-values to compare the performance of the proposed models for analysis. P-value values reflect the significance of statistical tests, and smaller p-values indicate more significant differences between the two models. For the RMSE metric, the p-value values of the AWD-stacking model were usually minimal (<0.001) compared to the other models on different prediction time ranges (30 min, 45 min, and 60 min). This implies that the AWD-stacking model has a significant advantage in terms of RMSE compared with other models, whether compared with BiLSTM, SLSTM, VLSTM, CBiLSTM, MABiLSTM, CBiLSTMA or BiLSTMA. This indicates that the AWD-stacking model can achieve better performance in predicting blood glucose levels. For the MAE metric, similar results to RMSE were observed. The p-value values of the AWD-stacking model were minimal (<0.001) compared to the other models on all data sets, which implies that the AWD-stacking model showed better performance in terms of MAE. The AWD-stacking model also shows significant advantages over different prediction time horizons for the MCC metric. The p-value values of the AWD-stacking model are all minimal (<0.001) compared to other models, indicating its better performance in terms of MCC. In conclusion, the AWD-stacking model showed a significant advantage over the other models in predicting blood glucose levels. It indicates that the AWD-stacking model is essential for blood glucose prediction in the T1DM dataset and is a more reliable and accurate model selection.

**Table 12:** P-values related to the post-hoc Wilcoxon test comparing all predictive models with each other on 12 datasets from the T1DM data contributors to RMSE.

PH	Model	BiLSTM	SLSTM	VLSTM	CBiLSTM	MABiLSTM	CBiLSTMA	BiLSTMA	AWD-stacking
30 min	BiLSTM	1.000	**<0.001**	**0.009**	0.077	0.129	**0.007**	**0.062**	**<0.001**
	SLSTM	**<0.001**	1.000	**<0.001**	**<0.001**	**<0.001**	**<0.001**	**<0.001**	**0.006**
	VLSTM	**0.009**	**<0.001**	1.000	**0.001**	**<0.001**	**<0.001**	**0.004**	**<0.001**
	CBiLSTM	**0.077**	**<0.001**	0.001	1.000	0.469	0.733	**0.042**	**<0.001**
	MABiLSTM	0.203	**<0.001**	**<0.001**	0.469	1.000	0.339	0.425	**<0.001**
	CBiLSTMA	**0.006**	**<0.001**	**<0.001**	0.733	0.339	1.000	0.109	**<0.001**
	BiLSTMA	0.622	**<0.001**	**0.004**	0.042	0.424	0.109	1.000	**<0.001**
	AWD-stacking	**<0.001**	**0.006**	**<0.001**	**<0.001**	**<0.001**	**<0.001**	**<0.001**	1.000
45min	BiLSTM	1.000	**<0.001**	**0.009**	0.569	0.622	0.622	0.969	**<0.001**
	SLSTM	**<0.001**	1.000	**<0.001**	**0.002**	**<0.001**	**<0.001**	**<0.001**	**<0.001**
	VLSTM	**0.009**	**<0.001**	1.000	0.092	0.034	0.077	0.176	**<0.001**
	CBiLSTM	0.569	**0.002**	0.092	1.000	0.969	0.791	0.204	**<0.001**
	MABiLSTM	0.622	**<0.001**	0.034	0.969	1.000	0.722	0.233	**<0.001**
	CBiLSTMA	0.622	**<0.001**	0.077	0.791	0.722	1.000	0.168	**<0.001**
	BiLSTMA	0.969	**<0.001**	0.176	0.203	0.233	0.168	1.000	**<0.001**
	AWD-stacking	**<0.001**	**<0.001**	**<0.001**	**<0.001**	**<0.001**	**<0.001**	**<0.001**	1.000
60min	BiLSTM	1.000	**<0.001**	**0.005**	0.235	0.568	0.684	0.841	**<0.001**
	SLSTM	**<0.001**	1.000	**<0.001**	**0.004**	**<0.001**	**<0.001**	**<0.001**	**<0.001**
	VLSTM	**0.005**	**<0.001**	1.000	**0.006**	0.064	0.089	0.049	**<0.001**
	CBiLSTM	0.235	0.004	0.006	1.000	0.591	0.764	0.092	**<0.001**
	MABiLSTM	0.568	**<0.001**	0.064	0.591	1.000	0.861	0.684	**<0.001**
	CBiLSTMA	0.684	**<0.001**	0.089	0.764	0.861	1.000	0.251	**<0.001**
	BiLSTMA	0.841	**<0.001**	0.049	0.092	0.684	0.251	1.000	**<0.001**
	AWD-stacking	**<0.001**	**<0.001**	**<0.001**	**<0.001**	**<0.001**	**<0.001**	**<0.001**	1.000

**Table 13:** P-values related to the post-hoc Wilcoxon test comparing all predictive models with each other on 12 datasets from the T1DM data contributors to MAE.

PH	Model	BiLSTM	SLSTM	VLSTM	CBiLSTM	MABiLSTM	CBiLSTMA	BiLSTMA	AWD-stacking
30 min	BiLSTM	1.000	**0.001**	**0.012**	0.569	0.006	**0.006**	0.176	**<0.001**
	SLSTM	**0.001**	1.000	**<0.001**	**<0.001**	**<0.001**	**<0.001**	**<0.001**	**<0.001**
	VLSTM	**0.012**	**<0.001**	1.000	**0.001**	**<0.001**	**<0.001**	**0.002**	**<0.001**
	CBiLSTM	0.569	**<0.001**	**0.001**	1.000	0.092	0.034	0.154	**<0.001**
	MABiLSTM	**0.006**	**<0.001**	**<0.001**	0.109	1.000	0.722	0.380	**<0.001**
	CBiLSTMA	**0.006**	**<0.001**	**<0.001**	0.034	0.722	1.000	0.622	**<0.001**
	BiLSTMA	0.176	**<0.001**	**0.002**	0.154	0.380	0.622	1.000	**<0.001**
	AWD-stacking	**<0.001**	**<0.001**	**<0.001**	**<0.001**	**<0.001**	**<0.001**	**<0.001**	1.000
45min	BiLSTM	1.000	**<0.001**	**0.091**	0.594	0.425	**0.005**	0.035	**<0.001**
	SLSTM	**<0.001**	1.000	**<0.001**	**0.005**	**<0.001**	**<0.001**	**<0.001**	**<0.001**
	VLSTM	0.091	**<0.001**	1.000	0.092	0.034	0.077	0.176	**<0.001**
	CBiLSTM	0.594	**0.005**	0.092	1.000	0.126	0.038	0.426	**<0.001**
	MABiLSTM	0.425	**<0.001**	0.034	0.126	1.000	0.688	0.235	**<0.001**
	CBiLSTMA	**0.005**	**<0.001**	0.077	0.038	0.688	1.000	0.654	**<0.001**
	BiLSTMA	0.035	**<0.001**	0.176	0.426	0.235	0.654	1.000	**<0.001**
	AWD-stacking	**<0.001**	**<0.001**	**<0.001**	**<0.001**	**<0.001**	**<0.001**	**<0.001**	1.000
60min	BiLSTM	1.000	**<0.001**	0.105	0.345	0.635	0.642	0.762	**<0.001**
	SLSTM	**<0.001**	1.000	**<0.001**	0.023	**<0.001**	**<0.001**	**<0.001**	**<0.001**
	VLSTM	0.105	**<0.001**	1.000	0.016	0.164	0.046	0.038	**<0.001**
	CBiLSTM	0.345	0.023	0.016	1.000	0.456	0.685	0.235	**<0.001**
	MABiLSTM	0.635	**<0.001**	0.164	0.456	1.000	0.562	0.624	**<0.001**
	CBiLSTMA	0.642	**<0.001**	0.046	0.685	0.562	1.000	0.253	**<0.001**
	BiLSTMA	0.762	**<0.001**	0.038	0.235	0.624	0.253	1.000	**<0.001**
	AWD-stacking	**<0.001**	**<0.001**	**<0.001**	**<0.001**	**<0.001**	**<0.001**	**<0.001**	1.000

**Table 14:** P-values related to the post-hoc Wilcoxon test comparing all predictive models with each other on 12 datasets from the T1DM data contributors to MCC.

PH	Model	BiLSTM	SLSTM	VLSTM	CBiLSTM	MABiLSTM	CBiLSTMA	BiLSTMA	AWD-stacking
30 min	BiLSTM	1.000	**0.026**	**<0.001**	<0.001	**0.003**	**0.012**	**0.026**	**<0.001**
	SLSTM	**0.026**	1.000	**0.034**	**0.677**	**0.109**	**0.050**	**0.092**	**<0.001**
	VLSTM	**<0.001**	**0.034**	1.000	0.021	**0.504**	**0.909**	**0.518**	**<0.001**
	CBiLSTM	**<0.001**	**0.677**	0.021	1.000	0.151	0.012	**0.021**	**<0.001**
	MABiLSTM	**0.003**	**0.109**	**0.504**	0.151	1.000	0.233	0.518	**<0.001**
	CBiLSTMA	**0.012**	**0.050**	**0.909**	0.012	0.233	1.000	0.850	**<0.001**
	BiLSTMA	0.026	**0.092**	0.518	0.021	0.518	0.850	1.000	**<0.001**
	AWD-stacking	**<0.001**	**<0.001**	**<0.001**	**<0.001**	**<0.001**	**<0.001**	**<0.001**	**<0.001**
45min	BiLSTM	1.000	**<0.001**	**0.031**	0.025	0.068	<0.001	0.035	**<0.001**
	SLSTM	**<0.001**	1.000	**<0.001**	0.015	**0.002**	**0.041**	**<0.001**	**<0.001**
	VLSTM	0.031	**<0.001**	1.000	0.025	0.065	0.684	0.876	**<0.001**
	CBiLSTM	0.025	**0.015**	0.025	1.000	0.969	0.791	0.204	**<0.001**
	MABiLSTM	0.068	**0.002**	0.065	0.969	1.000	0.354	0.495	**<0.001**
	CBiLSTMA	**<0.001**	**0.041**	0.684	0.791	0.354	1.000	0.234	**<0.001**
	BiLSTMA	0.035	**<0.001**	0.876	0.203	0.495	0.234	1.000	**<0.001**
	AWD-stacking	**<0.001**	**<0.001**	**<0.001**	**<0.001**	**<0.001**	**<0.001**	**<0.001**	1.000
60min	BiLSTM	1.000	**<0.001**	0.152	0.351	0.634	0.463	0.562	**<0.001**
	SLSTM	**<0.001**	1.000	**<0.001**	0.014	0.214	0.362	**<0.001**	**<0.001**
	VLSTM	0.152	**<0.001**	1.000	0.106	0.244	0.354	0.524	**<0.001**
	CBiLSTM	0.351	0.014	0.106	1.000	0.245	0.675	0.056	**<0.001**
	MABiLSTM	0.634	0.214	0.244	0.245	1.000	0.684	0.732	**<0.001**
	CBiLSTMA	0.463	0.362	0.354	0.675	0.684	1.000	0.451	**<0.001**
	BiLSTMA	0.562	**<0.001**	0.524	0.056	0.732	0.451	1.000	**<0.001**
	AWD-stacking	**<0.001**	**<0.001**	**<0.001**	**<0.001**	**<0.001**	**<0.001**	**<0.001**	1.000

## Discussion

This study aimed to explore the application of deep learning to BGL prediction. An Adaptive Weighted Decision Stacking ensemble learning model (AWD-stacking) was developed and validated using the OhioT1DM dataset. The proposed model achieves high accuracy in BGL prediction owing to several factors: i) the first application of the Kalman smoothing technique for BGL data preprocessing, which corrects data errors caused by sensor errors and improves model accuracy; ii) the use of double exponential smoothing for time-series data preprocessing to eliminate noise and outliers; iii) improved base estimator algorithms for BGL prediction yielding better results; iv) the utilization of an improved nearest neighbor propagation clustering algorithm for feature fusion, and increased model prediction accuracy; and v) the multi-historical window technique, which is proposed and applied to the AWD-stacking model.

Compared with other studies, the proposed algorithm demonstrated higher accuracy and practicality in BGL prediction. Table 15 compares state-of-the-art BGL prediction methods for the OhioT1DM clinical dataset. To ensure a fair comparison, this study used different versions of the ohiot1dm dataset with varying data volumes: (i) six subjects from the 2018 data and (ii) twelve subjects from the 2018 and 2020 data. Although some studies extend the PH to 120 minutes (corresponding to 24 data points), most relevant work only considers a 60-minute PH. Therefore, this study mainly focuses on comparisons with PH of 30 and 60 minutes, using RMSE and MAE as evaluation metrics.

**Table 15:** Comparison of different blood glucose prediction methods using RMSE and MAE as evaluation metrics.

	Authors	Methods	30-min PH		60-min PH	
			RMSE	MAE	RMSE	MAE
6 Subjects:2018 Dataset	Zhu T et al. [44]	CNN	21.72	-	-	-
	Midroni et al. [43]	XGBoost	20.377	-	-	-
	Li K et al. [47]	GluNet	19.28±2.76	-	31.83±3.49	-
	Zhu T et al. [20]	DRNN	19.04	-	-	-
	Şahin A et al. [45]	ANN	18.81	-	30.89	-
	Kang G et al. [46]	NPE+LSTM	17.8	-	-	-
	Rabby. F.M et al. [21]	StackLSTM	6.450	-	17.24	-
	-	**AWD-stacking**	**1.598**	**0.809**	**6.809**	**3.475**
12 Sub-jects 2018 Dataset and 2020 Dataset	Yang T et al. [24]	Auto-LSTM	18.930±2.155	-	-	-
	Zhu T et al. [48]	FCNN	18.64±2.60	-	31.07±3.62	-
	Martinsson J et al. [56]	RNN	18.867	-	31.403	-
	Shuvo M M H et al. [25]	DM-StackLSTM	16.06±2.74	10.64±4.10	30.89±4.31	22.07±2.96
	Butt H et al. [57]	Mult-LSTM	14.76	-	25.48	-
	Tena F et al. [23]	CE-DNN	19.57±3.03	14.06±2.15	34.93±5.29	25.95±3.61
	Daniels J et al. [49]	MTL-LSTM	18.8±2.3		31.8±3.9	
	Dudukcu et al. [22]	W-DLSTM	21.90	-	35.10	-
	Khadem et al. [50]	Nested-DE	23.74±0.15	13.48±0.02	34.35±0.86	27.76±0.38
	Kalita et al. [51]	LS-GRUNet	14.85	11.04	-	-
	Giacoma et al. [52]	LSTM-TCN	18.99	-	-	-
	Pavan et al. [53]	Shallow-Net	18.69	-	32.43	-
	Kim et al. [54]	RNN	21.50	-	-	-
	Freiburghaus et al. [55]	RCNN	17.45	11.22	33.67	23.25
	-	**AWD-stacking**	**1.425**	**0.721**	**6.346**	**3.232**

### 2018 dataset

In the dataset with six subjects in 2018, various machine learning algorithms for BGL prediction, such as XGBoost, were compared [43]. In addition, the proposed method is compared with state-of-the-art deep learning methods, including convolutional neural networks (CNN) [44], dilated recurrent neural networks (DRNN) [20], artificial neural networks (ANN) [45], stack long short-term memory (StackLSTM) [21], the fusion of neural physiological encoder (NPE) and long short-term memory (LSTM) [46], and an improved deep learning model for BGL prediction (GluNet) [47]. In the experiments conducted using the 2018 dataset with six subjects, the proposed model achieved the lowest RMSE and MAE for a 30-minute PH.

### 2018 and 2020 dataset

Twelve Subjects 2018 Dataset and 2020 Dataset: Validation using this dataset. In this study, the proposed development method was compared with the latest deep learning models., including the autonomous channel deep learning framework (Auto-LSTM) [24], fast-adaptive and confident neural network (FCNN) [48], deep multi-task stacked long short-term memory (DM-StackLSTM) [25], multi-layered long short-term memory (LSTM), cutting-edge deep neural networks (CE-DNN) [23], multi-task long short-term memory (MTL-LSTM) [49], Nested Deep Ensemble Learning (Nested-DE) [50], LS-GRUNet [51], long-short-term-memory and temporal convolutional networks(LSTM-TCN) [52], Shallow Network and Error Imputation(Shallow-Net) [53], recurrent neural network (RNN) [54], convolutional recurrent neural network (RCNN) [55] and weighted LSTM models (W-DLSTM) [22] applied to the experimental results of 12 subjects. The proposed method achieved the best results with the smallest root RMSE and MAE for 30 and 60 minutes PH. Overall, the proposed method outperformed those in the existing literature. When validated using the OhioT1DM dataset, the experimental results of the proposed algorithm were compared with those of the top-performing non-ensemble model, Stack-LSTM, as presented in Table 16. According to Tables 15 and 16 for a PH of 30 minutes, the proposed method achieves an RMSE of 1.425 mg/dL, an MAE of 0.721 mg/dL, and an MCC of 0.982 mg/dL. For a PH of 45 minutes, the RMSE was 3.212 mg/dL, the MAE was 1.605 mg/dL, and the MCC was 0.950 mg/dL. For a PH of 60 minutes, the RMSE was 6.346 mg/dL, MAE was 3.232 mg/dL, and MCC was 0.930 mg/dL. For all predictions, as the PH increased, the Matthews correlation coefficient (MCC) remained high, indicating a strong correlation between the prediction results of the model and the actual values and demonstrating good predictive performance.

**Table 16:** Prediction results of AWD-stacking and StackLSTM models.

Methods	Datasets	30-min PH		45-min PH		60-min PH	
		RMSE	MAE	RMSE	MAE	RMSE	MAE
AWD-stacking	2018	1.598	0.809	3.369	1.690	6.809	3.475
	2020	1.252	0.633	3.055	1.520	5.883	2.988
	**Average**	**1.425**	**0.721**	**3.212**	**1.605**	**6.346**	**3.232**
SLSTM	2018	2.001	1.259	3.789	2.271	7.555	4.252
	2020	1.645	1.125	3.512	2.188	6.484	4.277
	Average	**1.823**	**1.192**	**3.651**	**2.229**	**7.019**	**4.265**
An increase of X%	**Average**	**27.92%**	**65.32%**	**13.67%**	**38.87%**	**10.61%**	**31.96%**

Therefore, the proposed method demonstrated high accuracy and robustness in managing and predicting Type 1 diabetes BGLs. Furthermore, embedding this model in relevant medical devices for real-time on-site decision-making can effectively prevent adverse blood glucose events. The findings of this study have significant implications for managing patients with T1D, assisting doctors in decision-making, and improving patient quality of life.

## Conclusion

In treating diabetes patients, effective management of BGL concentrations and a deep understanding of BGLs are crucial. This paper proposes an adaptive algorithm based on deep ensemble learning for predicting BGLs, utilizing stacking ensemble learning with data preprocessing using Kalman filtering and double exponential smoothing. In time-series prediction, data smoothing has an essential effect on the prediction results because it reduces data noise and highlights the underlying patterns of the data. In this study, as shown in Fig 2, the results after smoothing (indicated in red in the figure) are better than the experimental results without smoothing (indicated in black in the figure), specifically, when using RMSE as the evaluation metric and a PH of 30 minutes, the smoothed prediction result is 1.425 mg/dL, and the non-smoothed result is 2.964 mg/dL, which is 51.92% higher. When RMSE and MAE were used as evaluation indicators, the smoothed results were 51.747% and 55.673% higher, respectively, than the average for the smoothed results. The results demonstrated that the results after smoothing were better than those without, using the evaluation indexes RMSE and MAE, and the error of the smoothed and unsmoothed data increased as the prediction horizon increased.

Effective management of BGL concentrations and insight into BGLs are crucial for the treatment of diabetic patients. This study proposes the use of an adaptive stacking ensemble learning method and compares it with seven non-integrated learning methods that help predict BGLs at 30, 45, and 60 min in advance. This study’s best non-ensemble models are the BiLSTM, StackLSTM, and VanilaLSTM models. These three non-ensembled models as the base learners of the ensemble model and the meta-learner is used to feature fuse the output of the base learners with adaptive weighting. Finally, the original training set features are fused to the training set of the meta-learner (the final training set input to the meta-learner contains three parts, the output of stacking ensemble learning, adaptive weighting of the three base learner outputs, and original training set). The features were fully learned to obtain accurate prediction results. After the experiments, the three non-ensemble models work best as the StackLSTM model, and we will use this non-ensemble model and the state-of-the-art models in the literature for comparison with the proposed integrated model. The multi-history window technique allows sufficient learning of data features, which is a multiple segmentation learning of historical datasets to better capture long-term dependencies and contextual information in time series data, which helps to improve the prediction results.

The study was a regression prediction. The RMSE, MAE, MCC and Critical difference diagrams were used to evaluate the experimental results. When the PH was 45 minutes, the RMSE, MAE and MCC were 3.212mg/dL,1.605mg/dL and 0.965mg/dL respectively. When the prediction range was 60 minutes, the RMSE, MAE and MCC were 6.346mg/dL,3.232mg/dL and 0.932mg/dL, respectively. The CDD plots show that the proposed algorithm provides the best prediction for all three PHs. Compared with the best non-ensemble model (SLSTM), the proposed model has improved RMSE, MAE and MCC. The results show that the developed model outperforms the best non-ensemble and state-of-the-art models in the literature, as shown in Table 15.

In this study, integrating the three best base learners into the model using the fault tolerance of ensemble learning resulted in a more accurate prediction. However, this study only used CGM data to build the BGL prediction model. In future work, it is recommended to consider the effects of multiple variables on blood glucose levels, such as sleep quality, carbohydrate intake, and insulin injection, and use the proposed method to predict BGL levels using a combination of multiple variables. Specifically, this study integrated data fusion techniques with the proposed method using multiple features added to the proposed model. In addition, tuning the hyperparameters of the integrated model can improve its accuracy. Finally, including other base learners and meta-learners in the examination would be valuable for future research.

## Supporting information

S1 Appendix Complete the multi-history window technique and detailed experimental results of 4 benchmarking models. (DOCX)
