# Supplementary material for: AWD-stacking: An enhanced ensemble learning model for predicting glucose levels
Source: PLoS One. 2024 Feb 14;19(2):e0291594. doi: 10.1371/journal.pone.0291594 (PMC10866533; doi:10.1371/journal.pone.0291594)
Supplement: S1 Appendix — (DOCX) [file pone.0291594.s001.docx]

Under the context of four historical window data, non-ensemble models were utilized to predict 12 patients and validate the performance of the non-ensemble models. For model evaluation, RMSE, MAE, and MCC metrics were employed to assess the predictive results of the models.

2018-Ⅰ:PH=30

| PID | Model | HW=30min | | | HW=45min | | | HW=60min | | | HW=90min | | |
| --- | --- | --- | --- | --- | --- | --- | --- | --- | --- | --- | --- | --- | --- |
|  |  | RMSE | MAE | MCC | RMSE | MAE | MCC | RMSE | MAE | MCC | RMSE | MAE | MCC |
| 559 | BiLSTM | 4.464 | 3.064 | 0.939 | 0.507 | 1.476 | 0.967 | 5.645 | 4.666 | 0.908 | 2.179 | 1.417 | 0.969 |
|  | SLSTM | 1.795 | 1.220 | 0.960 | 1.891 | 1.546 | 0.954 | 1.533 | 1.040 | 0.983 | 2.329 | 1.807 | 0.977 |
|  | VLSTM | 1.891 | 1.122 | 0.984 | 2.219 | 1.684 | 0.964 | 2.956 | 2.245 | 0.960 | 2.373 | 1.574 | 0.951 |
| 563 | BiLSTM | 7.003 | 4.714 | 0.915 | 8.552 | 6.342 | 0.902 | 2.501 | 1.470 | 0.980 | 2.017 | 1.307 | 0.980 |
|  | SLSTM | 2.177 | 1.415 | 0.974 | 1.980 | 1.812 | 0.975 | 1.882 | 0.985 | 0.980 | 1.856 | 0.981 | 0.980 |
|  | VLSTM | 3.974 | 2.442 | 0.959 | 3.271 | 1.997 | 0.969 | 2.604 | 1.645 | 0.973 | 2.681 | 1.611 | 0.973 |
| 570 | BiLSTM | 3.138 | 1.936 | 0.973 | 3.640 | 2.376 | 0.930 | 3.335 | 2.240 | 0.979 | 1.949 | 1.289 | 0.982 |
|  | SLSTM | 1.500 | 1.138 | 0.992 | 1.219 | 0.761 | 0.958 | 1.342 | 1.012 | 0.990 | 1.558 | 1.235 | 0.995 |
|  | VLSTM | 2.795 | 1.810 | 0.961 | 1.661 | 1.053 | 0.961 | 2.122 | 1.488 | 0.991 | 2.495 | 1.541 | 0.978 |
| 575 | BiLSTM | 5.460 | 3.386 | 0.947 | 4.100 | 2.352 | 0.931 | 4.951 | 3.237 | 0.956 | 2.560 | 1.362 | 0.976 |
|  | SLSTM | 2.524 | 1.390 | 0.985 | 2.261 | 1.131 | 0.949 | 2.366 | 1.193 | 0.985 | 2.355 | 1.120 | 0.988 |
|  | VLSTM | 2.815 | 1.418 | 0.978 | 2.695 | 1.358 | 0.946 | 3.289 | 1.880 | 0.983 | 3.026 | 1.597 | 0.976 |
| 588 | BiLSTM | 3.889 | 2.426 | 0.936 | 3.155 | 2.095 | 0.939 | 1.959 | 1.272 | 0.973 | 1.843 | 1.374 | 0.959 |
|  | SLSTM | 2.037 | 1.355 | 0.976 | 1.953 | 1.179 | 0.979 | 2.002 | 1.656 | 0.955 | 1.465 | 0.923 | 0.979 |
|  | VLSTM | 3.310 | 2.113 | 0.943 | 2.013 | 1.303 | 0.962 | 2.428 | 1.742 | 0.958 | 2.532 | 1.497 | 0.969 |
| 591 | BiLSTM | 4.515 | 3.029 | 0.904 | 5.358 | 3.662 | 0.887 | 4.031 | 2.561 | 0.934 | 2.952 | 0.850 | 0.954 |
|  | SLSTM | 2.252 | 1.549 | 0.967 | 2.055 | 1.166 | 0.972 | 2.031 | 1.243 | 0.962 | 2.033 | 1.238 | 0.966 |
|  | VLSTM | 3.519 | 2.149 | 0.952 | 2.444 | 1.429 | 0.968 | 2.533 | 1.529 | 0.966 | 2.905 | 1.762 | 0.953 |

2020-Ⅱ:PH=30

| PID | Model | HW=30min | | | HW=45min | | | HW=60min | | | HW=90min | | |
| --- | --- | --- | --- | --- | --- | --- | --- | --- | --- | --- | --- | --- | --- |
|  |  | RMSE | MAE | MCC | RMSE | MAE | MCC | RMSE | MAE | MCC | RMSE | MAE | MCC |
| 540 | BiLSTM | 8.395 | 7.138 | 0.810 | 4.641 | 3.063 | 0.917 | 2.211 | 1.448 | 0.967 | 1.545 | 0.961 | 0.973 |
|  | SLSTM | 1.441 | 1.404 | 0.969 | 1.278 | 0.857 | 0.982 | 1.225 | 0.782 | 0.983 | 1.234 | 0.759 | 0.982 |
|  | VLSTM | 3.551 | 2.460 | 0.940 | 2.013 | 1.330 | 0.963 | 1.489 | 0.894 | 0.974 | 1.662 | 1.066 | 0.974 |
| 544 | BiLSTM | 2.684 | 1.561 | 0.965 | 1.943 | 1.150 | 0.970 | 1.495 | 0.885 | 0.976 | 1.627 | 0.907 | 0.977 |
|  | SLSTM | 1.292 | 0.841 | 0.983 | 1.155 | 0.656 | 0.982 | 1.287 | 0.912 | 0.981 | 1.376 | 0.890 | 0.982 |
|  | VLSTM | 2.776 | 1.623 | 0.959 | 1.521 | 0.984 | 0.980 | 1.275 | 0.748 | 0.980 | 1.663 | 0.953 | 0.975 |
| 552 | BiLSTM | 3.324 | 2.277 | 0.955 | 2.811 | 2.257 | 0.959 | 4.161 | 3.328 | 0.972 | 1.470 | 1.107 | 0.983 |
|  | SLSTM | 1.323 | 0.792 | 0.986 | 1.321 | 0.856 | 0.985 | 1.059 | 0.648 | 0.992 | 1.386 | 0.970 | 0.992 |
|  | VLSTM | 3.751 | 2.263 | 0.961 | 1.561 | 0.873 | 0.985 | 1.384 | 0.925 | 0.984 | 2.031 | 1.236 | 0.986 |
| 567 | BiLSTM | 7.033 | 4.910 | 0.895 | 3.817 | 2.272 | 0.936 | 2.602 | 1.495 | 0.964 | 2.897 | 2.026 | 0.960 |
|  | SLSTM | 2.227 | 1.532 | 0.960 | 2.519 | 2.001 | 0.954 | 1.776 | 1.073 | 0.961 | 2.462 | 1.715 | 0.955 |
|  | VLSTM | 4.704 | 2.695 | 0.927 | 2.788 | 1.711 | 0.943 | 2.419 | 1.314 | 0.967 | 2.999 | 1.823 | 0.952 |
| 584 | BiLSTM | 4.249 | 2.580 | 0.953 | 3.315 | 1.921 | 0.954 | 6.142 | 4.783 | 0.873 | 2.865 | 1.661 | 0.950 |
|  | SLSTM | 2.650 | 1.420 | 0.964 | 2.482 | 1.987 | 0.961 | 2.401 | 1.682 | 0.955 | 1.994 | 1.240 | 0.965 |
|  | VLSTM | 4.706 | 3.150 | 0.906 | 2.506 | 1.448 | 0.960 | 3.194 | 1.983 | 0.930 | 2.588 | 1.517 | 0.959 |
| 596 | BiLSTM | 3.479 | 2.485 | 0.954 | 3.498 | 2.230 | 0.963 | 2.210 | 1.759 | 0.961 | 1.913 | 1.166 | 0.972 |
|  | SLSTM | 1.420 | 0.865 | 0.984 | 1.451 | 0.986 | 0.983 | 1.436 | 0.942 | 0.953 | 1.231 | 0.688 | 0.987 |
|  | VLSTM | 3.213 | 2.357 | 0.961 | 1.844 | 1.338 | 0.979 | 1.479 | 0.807 | 0.978 | 2.480 | 1.457 | 0.965 |

2018-Ⅰ:PH=45

| PID | Model | HW=30min | | | HW=45min | | | HW=60min | | | HW=90min | | |
| --- | --- | --- | --- | --- | --- | --- | --- | --- | --- | --- | --- | --- | --- |
|  |  | RMSE | MAE | MCC | RMSE | MAE | MCC | RMSE | MAE | MCC | RMSE | MAE | MCC |
| 559 | BiLSTM | 7.395 | 4.272 | 0.905 | 4.200 | 2.405 | 0.945 | 4.257 | 2.535 | 0.956 | 4.403 | 2.646 | 0.949 |
|  | SLSTM | 3.589 | 1.981 | 0.960 | 3.848 | 2.624 | 0.968 | 3.329 | 1.857 | 0.964 | 3.640 | 2.201 | 0.954 |
|  | VLSTM | 5.278 | 3.294 | 0.953 | 3.877 | 2.305 | 0.965 | 3.928 | 2.383 | 0.942 | 3.990 | 2.535 | 0.942 |
| 563 | BiLSTM | 6.641 | 3.951 | 0.927 | 7.285 | 4.420 | 0.908 | 4.518 | 2.582 | 0.956 | 4.656 | 2.625 | 0.950 |
|  | SLSTM | 4.632 | 2.950 | 0.960 | 4.168 | 2.091 | 0.964 | 4.213 | 2.091 | 0.972 | 4.184 | 2.163 | 0.961 |
|  | VLSTM | 7.075 | 4.473 | 0.954 | 4.787 | 2.689 | 0.949 | 5.051 | 2.709 | 0.942 | 5.699 | 3.265 | 0.935 |
| 570 | BiLSTM | 3.605 | 3.494 | 0.949 | 7.460 | 5.083 | 0.956 | 6.232 | 4.131 | 0.974 | 3.495 | 2.071 | 0.973 |
|  | SLSTM | 3.711 | 2.374 | 0.975 | 2.828 | 1.635 | 0.975 | 3.147 | 2.186 | 0.980 | 2.906 | 1.706 | 0.979 |
|  | VLSTM | 5.463 | 3.317 | 0.959 | 4.023 | 2.429 | 0.948 | 3.652 | 2.279 | 0.972 | 3.637 | 2.082 | 0.979 |
| 575 | BiLSTM | 11.841 | 7.317 | 0.899 | 7.099 | 4.296 | 0.934 | 6.827 | 3.981 | 0.961 | 5.975 | 3.127 | 0.944 |
|  | SLSTM | 6.714 | 4.507 | 0.952 | 6.101 | 3.337 | 0.953 | 5.656 | 2.827 | 0.956 | 5.447 | 2.985 | 0.959 |
|  | VLSTM | 11.300 | 6.960 | 0.899 | 7.117 | 3.943 | 0.947 | 6.638 | 3.578 | 0.937 | 8.309 | 5.266 | 0.924 |
| 588 | BiLSTM | 7.700 | 5.615 | 0.873 | 19.623 | 15.845 | 0.688 | 3.858 | 2.328 | 0.949 | 4.692 | 3.058 | 0.907 |
|  | SLSTM | 4.380 | 2.613 | 0.946 | 3.717 | 2.968 | 0.954 | 3.760 | 2.160 | 0.947 | 3.530 | 2.054 | 0.952 |
|  | VLSTM | 5.822 | 3.355 | 0.917 | 4.273 | 2.779 | 0.922 | 4.723 | 2.722 | 0.939 | 4.319 | 2.459 | 0.933 |
| 591 | BiLSTM | 9.367 | 5.825 | 0.853 | 10.503 | 6.923 | 0.828 | 6.574 | 4.005 | 0.893 | 5.957 | 3.724 | 0.906 |
|  | SLSTM | 4.729 | 2.695 | 0.932 | 4.623 | 2.641 | 0.943 | 4.583 | 2.726 | 0.940 | 4.843 | 2.950 | 0.939 |
|  | VLSTM | 9.066 | 5.679 | 0.935 | 6.538 | 3.871 | 0.903 | 5.658 | 3.232 | 0.916 | 6.926 | 4.009 | 0.900 |

2020-Ⅱ:PH=45

| PID | Model | HW=30min | | | HW=45min | | | HW=60min | | | HW=90min | | |
| --- | --- | --- | --- | --- | --- | --- | --- | --- | --- | --- | --- | --- | --- |
|  |  | RMSE | MAE | MCC | RMSE | MAE | MCC | RMSE | MAE | MCC | RMSE | MAE | MCC |
| 540 | BiLSTM | 9.549 | 6.687 | 0.824 | 5.029 | 3.079 | 0.896 | 3.273 | 1.926 | 0.945 | 3.279 | 1.971 | 0.945 |
|  | SLSTM | 3.418 | 2.139 | 0.951 | 3.349 | 1.917 | 0.951 | 2.791 | 1.561 | 0.955 | 2.772 | 1.540 | 0.954 |
|  | VLSTM | 5.116 | 3.541 | 0.911 | 4.324 | 2.602 | 0.926 | 3.407 | 2.079 | 0.936 | 3.857 | 2.383 | 0.936 |
| 544 | BiLSTM | 4.918 | 2.839 | 0.926 | 4.185 | 2.544 | 0.940 | 3.359 | 2.217 | 0.955 | 2.860 | 1.505 | 0.967 |
|  | SLSTM | 2.920 | 1.955 | 0.960 | 3.580 | 2.262 | 0.971 | 2.608 | 1.563 | 0.965 | 2.339 | 1.249 | 0.973 |
|  | VLSTM | 3.211 | 1.836 | 0.961 | 3.875 | 2.287 | 0.948 | 2.900 | 1.589 | 0.965 | 3.622 | 1.839 | 0.960 |
| 552 | BiLSTM | 6.628 | 4.424 | 0.921 | 4.458 | 2.274 | 0.953 | 3.080 | 1.602 | 0.980 | 3.438 | 2.102 | 0.969 |
|  | SLSTM | 3.010 | 1.653 | 0.976 | 3.584 | 2.923 | 0.966 | 2.965 | 2.105 | 0.976 | 2.585 | 1.350 | 0.978 |
|  | VLSTM | 6.029 | 3.724 | 0.948 | 3.399 | 2.158 | 0.979 | 3.378 | 1.937 | 0.975 | 3.427 | 2.009 | 0.978 |
| 567 | BiLSTM | 10.503 | 6.828 | 0.868 | 7.250 | 4.110 | 0.893 | 4.889 | 2.898 | 0.911 | 4.007 | 2.242 | 0.941 |
|  | SLSTM | 5.064 | 3.213 | 0.894 | 4.614 | 2.825 | 0.914 | 4.354 | 2.391 | 0.927 | 3.442 | 2.278 | 0.953 |
|  | VLSTM | 9.451 | 5.390 | 0.871 | 6.418 | 3.942 | 0.888 | 6.107 | 3.432 | 0.927 | 4.504 | 2.620 | 0.938 |
| 584 | BiLSTM | 8.547 | 5.251 | 0.886 | 5.174 | 3.321 | 0.925 | 5.128 | 2.976 | 0.934 | 4.773 | 2.699 | 0.935 |
|  | SLSTM | 4.962 | 3.035 | 0.929 | 5.103 | 3.109 | 0.921 | 4.639 | 2.734 | 0.930 | 4.737 | 2.941 | 0.930 |
|  | VLSTM | 5.610 | 3.231 | 0.902 | 6.055 | 3.506 | 0.927 | 4.765 | 2.655 | 0.938 | 6.964 | 4.764 | 0.864 |
| 596 | BiLSTM | 5.675 | 3.352 | 0.941 | 6.239 | 4.190 | 0.933 | 3.581 | 1.919 | 0.966 | 3.686 | 2.381 | 0.953 |
|  | SLSTM | 3.257 | 2.094 | 0.958 | 3.247 | 2.121 | 0.970 | 3.226 | 1.913 | 0.974 | 2.916 | 1.634 | 0.971 |
|  | VLSTM | 5.776 | 3.632 | 0.924 | 2.562 | 2.024 | 0.963 | 3.981 | 2.362 | 0.969 | 4.220 | 2.360 | 0.951 |
|  | EModel | 2.743 | 1.370 | 0.972 | 2.726 | 1.354 | 0.972 | 2.756 | 1.378 | 0.976 | 2.775 | 1.388 | 0.970 |

2018-Ⅰ:PH=60

| PID | Model | HW=30min | | | HW=45min | | | HW=60min | | | HW=90min | | |
| --- | --- | --- | --- | --- | --- | --- | --- | --- | --- | --- | --- | --- | --- |
|  |  | RMSE | MAE | MCC | RMSE | MAE | MCC | RMSE | MAE | MCC | RMSE | MAE | MCC |
| 559 | BiLSTM | 12.965 | 9.207 | 0.756 | 8.215 | 4.585 | 0.917 | 6.807 | 4.244 | 0.931 | 7.585 | 4.896 | 0.920 |
|  | SLSTM | 6.513 | 3.847 | 0.930 | 6.342 | 3.390 | 0.888 | 6.089 | 3.393 | 0.942 | 6.884 | 3.922 | 0.942 |
|  | VLSTM | 12.691 | 7.937 | 0.877 | 7.540 | 4.274 | 0.924 | 8.205 | 5.218 | 0.872 | 8.030 | 5.887 | 0.887 |
| 563 | BiLSTM | 13.497 | 8.589 | 0.851 | 8.038 | 4.500 | 0.924 | 8.251 | 5.115 | 0.909 | 8.616 | 5.041 | 0.902 |
|  | SLSTM | 8.137 | 4.488 | 0.931 | 7.507 | 3.891 | 0.912 | 7.705 | 4.204 | 0.937 | 7.592 | 4.034 | 0.931 |
|  | VLSTM | 10.500 | 6.800 | 0.869 | 8.949 | 5.476 | 0.898 | 8.985 | 5.054 | 0.915 | 9.910 | 5.152 | 0.906 |
| 570 | BiLSTM | 8.778 | 5.400 | 0.930 | 11.303 | 7.896 | 0.921 | 7.355 | 4.631 | 0.947 | 7.743 | 4.762 | 0.954 |
|  | SLSTM | 6.719 | 4.161 | 0.943 | 5.781 | 3.381 | 0.912 | 4.996 | 2.750 | 0.957 | 5.104 | 2.796 | 0.948 |
|  | VLSTM | 8.583 | 5.187 | 0.929 | 6.960 | 4.069 | 0.943 | 7.534 | 4.957 | 0.952 | 5.926 | 3.201 | 0.943 |
| 575 | BiLSTM | 10.084 | 5.179 | 0.916 | 10.957 | 5.618 | 0.914 | 14.089 | 8.022 | 0.879 | 11.327 | 5.653 | 0.900 |
|  | SLSTM | 9.767 | 5.359 | 0.913 | 9.600 | 4.993 | 0.923 | 9.767 | 4.738 | 0.929 | 10.624 | 5.994 | 0.917 |
|  | VLSTM | 14.516 | 8.042 | 0.855 | 10.307 | 5.336 | 0.914 | 10.620 | 5.300 | 0.906 | 11.509 | 5.913 | 0.894 |
| 588 | BiLSTM | 13.293 | 9.440 | 0.802 | 11.888 | 7.324 | 0.828 | 7.935 | 4.928 | 0.899 | 7.926 | 4.636 | 0.892 |
|  | SLSTM | 7.075 | 3.927 | 0.917 | 7.209 | 4.535 | 0.898 | 6.399 | 3.796 | 0.924 | 6.443 | 3.627 | 0.926 |
|  | VLSTM | 8.807 | 5.120 | 0.870 | 7.143 | 4.151 | 0.902 | 7.015 | 3.966 | 0.907 | 7.399 | 4.432 | 0.899 |
| 591 | BiLSTM | 14.895 | 10.259 | 0.775 | 14.337 | 9.211 | 0.799 | 10.283 | 6.072 | 0.872 | 9.944 | 6.101 | 0.860 |
|  | SLSTM | 10.121 | 5.938 | 0.884 | 8.024 | 4.423 | 0.897 | 8.776 | 5.566 | 0.899 | 8.130 | 4.898 | 0.884 |
|  | VLSTM | 13.277 | 8.018 | 0.819 | 11.382 | 7.085 | 0.811 | 10.089 | 6.289 | 0.858 | 9.843 | 5.643 | 0.875 |

2020-Ⅱ:PH=60

| PID | Model | HW=30min | | | HW=45min | | | HW=60min | | | HW=90min | | |
| --- | --- | --- | --- | --- | --- | --- | --- | --- | --- | --- | --- | --- | --- |
|  |  | RMSE | MAE | MCC | RMSE | MAE | MCC | RMSE | MAE | MCC | RMSE | MAE | MCC |
| 540 | BiLSTM | 11.568 | 7.851 | 0.772 | 8.003 | 4.944 | 0.855 | 6.551 | 3.734 | 0.899 | 6.641 | 4.113 | 0.878 |
|  | SLSTM | 6.942 | 4.391 | 0.883 | 5.784 | 3.776 | 0.898 | 5.898 | 3.752 | 0.902 | 5.524 | 3.250 | 0.909 |
|  | VLSTM | 12.351 | 8.358 | 0.761 | 6.199 | 3.724 | 0.890 | 5.706 | 3.341 | 0.902 | 6.969 | 4.241 | 0.874 |
| 544 | BiLSTM | 7.692 | 4.291 | 0.880 | 6.734 | 3.750 | 0.915 | 7.447 | 4.560 | 0.884 | 5.667 | 3.068 | 0.923 |
|  | SLSTM | 5.176 | 2.700 | 0.927 | 5.240 | 3.409 | 0.927 | 4.800 | 2.530 | 0.939 | 4.771 | 2.580 | 0.937 |
|  | VLSTM | 8.533 | 5.148 | 0.874 | 5.368 | 3.049 | 0.928 | 6.059 | 3.700 | 0.914 | 6.208 | 3.386 | 0.917 |
| 552 | BiLSTM | 9.634 | 5.839 | 0.895 | 6.968 | 3.415 | 0.920 | 5.609 | 2.591 | 0.943 | 6.390 | 3.317 | 0.940 |
|  | SLSTM | 6.038 | 3.434 | 0.958 | 5.184 | 2.967 | 0.952 | 4.963 | 2.576 | 0.956 | 4.898 | 2.916 | 0.957 |
|  | VLSTM | 8.845 | 4.978 | 0.904 | 5.168 | 2.435 | 0.957 | 5.978 | 2.955 | 0.947 | 6.654 | 3.403 | 0.935 |
| 567 | BiLSTM | 15.045 | 9.050 | 0.765 | 12.740 | 7.945 | 0.836 | 9.513 | 5.121 | 0.909 | 11.372 | 7.171 | 0.876 |
|  | SLSTM | 9.689 | 5.733 | 0.876 | 8.086 | 4.210 | 0.909 | 8.214 | 5.185 | 0.884 | 8.630 | 4.502 | 0.917 |
|  | VLSTM | 10.055 | 5.717 | 0.870 | 11.004 | 6.134 | 0.899 | 10.785 | 5.841 | 0.870 | 11.441 | 6.303 | 0.870 |
| 584 | BiLSTM | 15.996 | 11.093 | 0.690 | 15.960 | 10.977 | 0.762 | 8.853 | 4.892 | 0.889 | 9.640 | 5.791 | 0.874 |
|  | SLSTM | 9.462 | 6.284 | 0.861 | 8.671 | 4.868 | 0.845 | 8.311 | 4.609 | 0.882 | 8.506 | 5.120 | 0.885 |
|  | VLSTM | 13.621 | 8.858 | 0.761 | 12.519 | 8.240 | 0.812 | 9.938 | 5.691 | 0.840 | 12.040 | 7.024 | 0.823 |
| 596 | BiLSTM | 9.410 | 5.826 | 0.907 | 10.998 | 7.626 | 0.870 | 6.077 | 3.902 | 0.937 | 6.492 | 3.804 | 0.935 |
|  | SLSTM | 5.438 | 3.127 | 0.949 | 5.423 | 3.210 | 0.946 | 5.308 | 3.107 | 0.950 | 5.508 | 3.139 | 0.947 |
|  | VLSTM | 8.042 | 4.718 | 0.920 | 6.324 | 3.548 | 0.942 | 5.932 | 3.216 | 0.942 | 6.090 | 3.377 | 0.939 |

Prediction results of 4 benchmarking models with AWD-stacking for 6 patients (in 2018 data)

| PID | Model | PH=30min | | | PH=45min | | | PH=60min | | |
| --- | --- | --- | --- | --- | --- | --- | --- | --- | --- | --- |
|  |  | RMSE | MAE | MCC | RMSE | MAE | MCC | RMSE | MAE | MCC |
| 559 | CBiLSTM | 3.658 | 2.361 | 0.954 | 5.623 | 2.864 | 0.951 | 9.681 | 5.621 | 0.942 |
|  | MABiLSTM | 4.658 | 3.954 | 0.962 | 6.284 | 4.678 | 0.958 | 9.824 | 6.325 | 0.936 |
|  | CBiLSTMA | 4.759 | 3.215 | 0.953 | 7.596 | 5.241 | 0.935 | 10.832 | 7.659 | 0.928 |
|  | BiLSTMA | 3.954 | 2.685 | 0.961 | 4.975 | 4.268 | 0.953 | 7.924 | 5.674 | 0.945 |
|  | AWD-stacking | 1.285 | 0.896 | 0.988 | 3.168 | 1.562 | 0.968 | 5.966 | 3.011 | 0.951 |
| 563 | CBiLSTM | 4.569 | 2.598 | 0.962 | 5.601 | 3.635 | 0..951 | 7.589 | 5.601 | 0.942 |
|  | MABiLSTM | 5.692 | 4.036 | 0.975 | 7.352 | 4.369 | 0.965 | 9.364 | 6.251 | 0.952 |
|  | CBiLSTMA | 5.284 | 3.184 | 0.963 | 7.982 | 4.265 | 0.953 | 10.695 | 5.261 | 0.949 |
|  | BiLSTMA | 4.025 | 2.695 | 0.958 | 6.205 | 3.251 | 0.949 | 8.019 | 6.384 | 0.938 |
|  | AWD-stacking | 1.735 | 0.792 | 0.981 | 3.988 | 1.897 | 0.964 | 7.166 | 3.526 | 0.933 |
| 570 | CBiLSTM | 5.695 | 2.695 | 0.975 | 7.399 | 3.695 | 0.965 | 9.285 | 4.698 | 0.952 |
|  | MABiLSTM | 6.325 | 4.581 | 0.962 | 7.851 | 5.841 | 0.958 | 10.396 | 6.281 | 0.935 |
|  | CBiLSTMA | 4.265 | 3.368 | 0.968 | 6.584 | 5.984 | 0.936 | 8.028 | 6.981 | 0.925 |
|  | BiLSTMA | 4.695 | 3.987 | 0.972 | 6.981 | 5.698 | 0.957 | 9.587 | 7.295 | 0.948 |
|  | AWD-stacking | 1.239 | 0.608 | 0.990 | 2.702 | 1.369 | 0.985 | 5.044 | 2.595 | 0.957 |
| 575 | CBiLSTM | 4.395 | 3.265 | 0.985 | 6.325 | 4.362 | 0.974 | 10.685 | 5.682 | 0.963 |
|  | MABiLSTM | 3.685 | 3.362 | 0.976 | 5.625 | 4.265 | 0.965 | 10.036 | 6.258 | 0.964 |
|  | CBiLSTMA | 4.285 | 3.251 | 0.968 | 5.326 | 5.263 | 0.952 | 9.362 | 7.263 | 0.935 |
|  | BiLSTMA | 3.698 | 2.621 | 0.958 | 4.652 | 3.625 | 0.946 | 11.652 | 6.325 | 0.942 |
|  | AWD-stacking | 2.184 | 0.942 | 0.977 | 2.702 | 1.369 | 0.985 | 9.487 | 4.400 | 0.921 |
| 588 | CBiLSTM | 5.265 | 3.658 | 0.976 | 7.635 | 4.265 | 0.965 | 8.365 | 5.684 | 0.945 |
|  | MABiLSTM | 3.265 | 2.695 | 0.958 | 5.236 | 3.268 | 0.945 | 7.365 | 6.328 | 0.956 |
|  | CBiLSTMA | 3.685 | 3.284 | 0.968 | 5.263 | 4.698 | 0.958 | 6.358 | 7.265 | 0.942 |
|  | BiLSTMA | 4.698 | 3.981 | 0.965 | 6.325 | 5.751 | 0.948 | 7.268 | 6.325 | 0.934 |
|  | AWD-stacking | 1.316 | 0.703 | 0.979 | 3.272 | 1.719 | 0.958 | 5.257 | 3.168 | 0.929 |
| 591 | CBiLSTM | 4.369 | 2.395 | 0.968 | 6.287 | 4.659 | 0.938 | 8.369 | 5.694 | 0.891 |
|  | MABiLSTM | 5.326 | 3.358 | 0.958 | 6.847 | 5.364 | 0.924 | 9.185 | 6.584 | 0.881 |
|  | CBiLSTMA | 4.851 | 4.587 | 0.957 | 7.695 | 6.328 | 0.938 | 8.384 | 7.394 | 0.873 |
|  | BiLSTMA | 3.269 | 4.698 | 0.949 | 5.694 | 5.948 | 0.927 | 8.691 | 6.284 | 0.869 |
|  | AWD-stacking | 1.834 | 0.917 | 0.974 | 4.380 | 2.226 | 0.947 | 7.935 | 4.148 | 0.903 |
| AVG | CBiLSTM | 4.658 | 2.828 | 0.970 | 6.478 | 3.913 | 0.958 | 8.996 | 5.497 | 0.939 |
|  | MABiLSTM | 4.825 | 3.664 | 0.965 | 6.533 | 4.631 | 0.953 | 9.361 | 6.337 | 0.937 |
|  | CBiLSTMA | 4.521 | 3.482 | 0.962 | 6.008 | 5.297 | 0.945 | 8.943 | 6.971 | 0.925 |
|  | BiLSTMA | 4.056 | 3.652 | 0.960 | 5.504 | 4.757 | 0.946 | 8.856 | 6.665 | 0.929 |
|  | AWD-stacking | **1.598** | **0.809** | **0.982** | **3.369** | **1.690** | **0.968** | **6.809** | **3.475** | **0.932** |

Prediction results of 4 benchmarking models with AWD-stacking for 6 patients (in 2020 data)

| PID | Model | PH=30min | | | PH=45min | | | PH=60min | | |
| --- | --- | --- | --- | --- | --- | --- | --- | --- | --- | --- |
|  |  | RMSE | MAE | MCC | RMSE | MAE | MCC | RMSE | MAE | MCC |
| 540 | CBiLSTM | 5.694 | 3.684 | 0.965 | 7.329 | 4.684 | 0.946 | 8.954 | 5.698 | 0.901 |
|  | MABiLSTM | 4.268 | 3.594 | 0.975 | 6.325 | 5.021 | 0.948 | 9.328 | 6.328 | 0.894 |
|  | CBiLSTMA | 5.384 | 4.274 | 0.974 | 6.684 | 5.628 | 0.933 | 8.217 | 7.268 | 0.888 |
|  | BiLSTMA | 4.268 | 3.684 | 0.965 | 5.298 | 4.395 | 0.922 | 7.295 | 5.128 | 0.908 |
|  | AWD-stacking | 1.046 | 0.552 | 0.984 | 2.683 | 1.419 | 0.955 | 5.140 | 2.772 | 0.916 |
| 544 | CBiLSTM | 4.378 | 3.244 | 0.975 | 6.325 | 5.321 | 0.956 | 7.974 | 6.698 | 0.924 |
|  | MABiLSTM | 3.458 | 3.245 | 0.945 | 7.148 | 4.658 | 0.957 | 9.368 | 6.456 | 0.914 |
|  | CBiLSTMA | 4.384 | 4.658 | 0.964 | 6.385 | 5.268 | 0.936 | 8.235 | 7.785 | 0.925 |
|  | BiLSTMA | 4.865 | 3.265 | 0.955 | 5.298 | 4.978 | 0.954 | 7.98 | 6.328 | 0.938 |
|  | AWD-stacking | 0.992 | 0.552 | 0.984 | 2.374 | 1.167 | 0.974 | 4.569 | 2.260 | 0.941 |
| 552 | CBiLSTM | 5.329 | 2.358 | 0.985 | 6.384 | 3.654 | 0.975 | 7.395 | 4.685 | 0.942 |
|  | MABiLSTM | 2.358 | 3.285 | 0.967 | 5.584 | 4.698 | 0.968 | 6.328 | 5.268 | 0.935 |
|  | CBiLSTMA | 4.256 | 2.687 | 0.935 | 3.265 | 3.759 | 0.984 | 4.265 | 4.268 | 0.928 |
|  | BiLSTMA | 3.985 | 3.387 | 0.975 | 4.268 | 4.659 | 0.962 | 8.295 | 7.685 | 0.925 |
|  | AWD-stacking | 0.935 | 0.409 | 0.994 | 2.385 | 1.033 | 0.980 | 4.631 | 2.071 | 0.955 |
| 567 | CBiLSTM | 2.658 | 1.285 | 0.964 | 3.685 | 2.685 | 0.936 | 8.698 | 5.326 | 0.891 |
|  | MABiLSTM | 3.268 | 1.974 | 0.968 | 5.384 | 3.268 | 0.952 | 8.235 | 4.526 | 0.905 |
|  | CBiLSTMA | 4.295 | 2.658 | 0.957 | 5.268 | 3.584 | 0.937 | 7.965 | 5.284 | 0.916 |
|  | BiLSTMA | 2.385 | 1.357 | 0.972 | 4.285 | 2.785 | 0.948 | 7.284 | 4.511 | 0.885 |
|  | AWD-stacking | 1.570 | 0.788 | 0.979 | 3.752 | 1.877 | 0.954 | 7.802 | 3.963 | 0.919 |
| 584 | CBiLSTM | 3.251 | 2.385 | 0.962 | 4.685 | 4.658 | 0.925 | 9.328 | 6.328 | 0.865 |
|  | MABiLSTM | 4.256 | 3.658 | 0.958 | 5.628 | 4.328 | 0.935 | 8.574 | 5.368 | 0.847 |
|  | CBiLSTMA | 3.265 | 2.365 | 0.948 | 4.958 | 4.168 | 0.914 | 8.694 | 6.385 | 0.883 |
|  | BiLSTMA | 4.283 | 2.584 | 0.935 | 7.368 | 3.684 | 0.905 | 9.158 | 7.258 | 0.872 |
|  | AWD-stacking | 1.826 | 0.936 | 0.970 | 4.388 | 2.253 | 0.939 | 8.083 | 4.205 | 0.885 |
| 596 | CBiLSTM | 4.328 | 3.251 | 0.974 | 4.698 | 4.625 | 0.956 | 5.685 | 5.268 | 0.925 |
|  | MABiLSTM | 2.658 | 1.285 | 0.965 | 3.658 | 3.158 | 0.945 | 6.682 | 4.362 | 0.932 |
|  | CBiLSTMA | 3.157 | 2.695 | 0.964 | 4.591 | 4.268 | 0.958 | 6.012 | 5.261 | 0.914 |
|  | BiLSTMA | 2.018 | 1.394 | 0.976 | 5.261 | 3.685 | 0.964 | 5.681 | 4.362 | 0.925 |
|  | AWD-stacking | 1.147 | 0.558 | 0.987 | 2.750 | 1.372 | 0.972 | 5.070 | 2.661 | 0.954 |
| AVG | CBiLSTM | 4.273 | 2.701 | 0.970 | 5.517 | 4.271 | 0.949 | 8.005 | 5.667 | 0.908 |
|  | MABiLSTM | 3.377 | 2.840 | 0.963 | 5.621 | 4.188 | 0.951 | 8.081 | 5.385 | 0.904 |
|  | CBiLSTMA | 4.124 | 3.222 | 0.957 | 5.191 | 4.445 | 0.943 | 7.231 | 6.042 | 0.909 |
|  | BiLSTMA | 3.634 | 2.611 | 0.963 | 5.296 | 4.031 | 0.942 | 7.615 | 5.878 | 0.908 |
|  | AWD-stacking | **1.252** | **0.633** | **0.983** | **3.055** | **1.520** | **0.962** | **5.883** | **2.988** | **0.928** |
